# Transcriptomic evidence for the control of soybean root isoflavonoid content by regulation of overlapping phenylpropanoid pathways

**DOI:** 10.1186/s12864-016-3463-y

**Published:** 2017-01-11

**Authors:** Mehran Dastmalchi, Patrick Chapman, Jaeju Yu, Ryan S. Austin, Sangeeta Dhaubhadel

**Affiliations:** 1 Department of Biology, University of Western Ontario, London, ON Canada; 2 London Research and Development Centre, Agriculture and Agri-Food Canada, 1391 Sandford Street, London, ON N5V 4T3 Canada

**Keywords:** Soybean, Secondary metabolism, Phenylpropanoid, Isoflavonoid, Root, Transcriptome, RNA sequencing

## Abstract

**Background:**

Isoflavonoids are a class of specialized metabolites found predominantly in legumes. They play a role in signaling for symbiosis with nitrogen-fixing bacteria and inhibiting pathogen infection.

**Results:**

A transcriptomic approach using soybean cultivars with high (Conrad and AC Colombe) and low (AC Glengarry and Pagoda) root isoflavonoid content was used to find elements that underlie this variation. Two genes, encoding the flavonoid-metabolizing enzymes, flavonoid 3′-hydroxylase (*GmF3′H*) and dihydroflavonol 4-reductase (*GmDFR*)*,* had lower expression levels in high isoflavonoid cultivars. These enzymes compete with isoflavonoid biosynthetic enzymes for the important branch-point substrate naringenin and its derivatives. Differentially expressed genes, between the two sets of cultivars, encode transcription factors, transporters and enzymatic families of interest, such as oxidoreductases, hydrolases and transferases. In addition, genes annotated with stress and disease response were upregulated in high isoflavonoid cultivars.

**Conclusions:**

Coordinated regulation of genes involved in flavonoid metabolism could redirect flux into the isoflavonoid branch of the phenylpropanoid pathway, by reducing competition for the flavanone substrate. These candidate genes could help identify mechanisms to overcome the endogenous bottleneck to isoflavonoid production, facilitate biosynthesis in heterologous systems, and enhance crop resistance against pathogenic infections.

**Electronic supplementary material:**

The online version of this article (doi:10.1186/s12864-016-3463-y) contains supplementary material, which is available to authorized users.

## Background

Soybean is a paleopolyploid with two duplication events, approximately 14 and 44 million years ago [1]. The majority of phenylpropanoid and specialized metabolism enzymes that have been characterized belong to multi-gene families [2–4]. Functional divergence within these families have been conjectured to be partially responsible for the array of plant specialized metabolites [5].

In the phenylpropanoid pathway chalcone synthase (CHS) produces the chalcone nucleus that is then utilized in downstream metabolite biosynthesis. There are at least nine *CHS* genes in soybean (*GmCHS1-GmCHS9*) that share 89.43 to 99.48% sequence identity at the amino acid level [6]. Members of the *CHS* gene family are differentially expressed, respond to different stimuli, and have shown functional divergence. *GmCHS7* and *GmCHS8* are critical for isoflavonoid biosynthesis and accumulation in soybean seeds [7]. These two enzymes have shown differential localization; GmCHS7 was localized to the cytoplasm, while GmCHS8 was localized to the nucleus and cytoplasm [8]. The level of differentiation in the expression of almost identical genes in the *CHS* family, and the putative functional specialization of their cognate proteins underlines the complexity associated with multi-gene families in large genomes such as soybean. Genetic and functional variation have also been evidenced in other closely linked enzyme families such as chalcone isomerase (CHI) [9, 10], chalcone reductase (CHR) [11–13] and isoflavonoid transferases [14].

The characterization of multi-gene families is important in the study of overlapping branches in the phenylpropanoid pathway. Specialization can explain mechanisms that mediate competition between shared enzymes and metabolites. Further, it can describe the evolutionary path that leads to new legume or species-specific metabolites such as isoflavonoids. The competition between the flavonoid and isoflavonoid branches of the phenylpropanoid pathway has been described in the shared usage of flavanone substrate, naringenin, and enzymes, such as CHS and CHI [15]. The evolution of enzymatic capacity is apparent in the evolution of the CHI fold from protist homologs to the legume-specific catalysis of isoliquiritigenin and naringenin chalcone to liquiritigenin and naringenin, respectively [9].

**Fig. 1:**
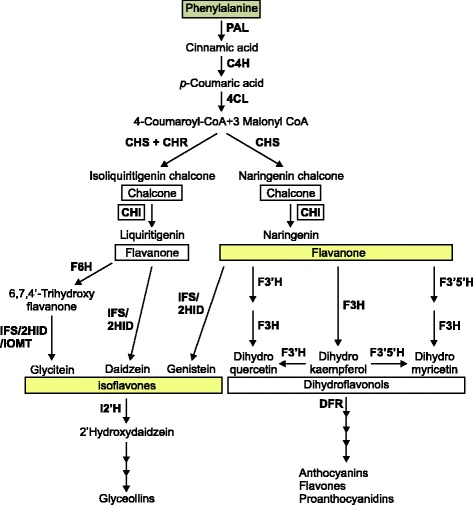
Phenylalanine recruited into a diverse network of metabolism including the production of isoflavones and other specialized metabolites. Competition for the common substrate naringenin is highlighted, with downstream metabolites in yellow. Chalcone synthase (CHS) produces the chalcone, naringenin chalcone, and in legumes CHS coactively with chalcone reductase (CHR) forms isoliquiritigenin chalcone. Chalcone isomerase (CHI) converts the two chalcones to their flavanone derivatives: liquiritigenin and naringenin; the latter, as stated above, can be utilized by isoflavones synthase (IFS) or a whole host of other enzymes, to produce a variety of metabolites including the isoflavones. The multiple arrows indicate two or more steps in the pathway. Other enzymes labeled: PAL, phenylalanine ammonia lyase; C4H, cinnamate 4-hydroxylase; 4CL, 4-coumarate:CoA ligase; 2HID, 2-Hydroxyisoflavanone dehydratase; IOMT, isoflavone O-methyltransferase; F6H, flavonoid 6-hydroxylase

Isoflavonoid biosynthesis is a legume-specific branch of the diverse phenylpropanoid pathway (Fig. 1). This class of specialized metabolites is involved in pathogen inhibition and nitrogen-fixing symbiosis [16–19]. As part of human diet isoflavonoids are linked with a reduction in the risk of cardiovascular disease and hormone-dependent cancers [20–23]. The isoflavonoid pathway is in direct competition with the concurrent flavonoid pathways for flavanone substrates. Efforts to manipulate isoflavonoid biosynthesis or to engineer the pathway in non-legumes have underlined this effective bottleneck to metabolite accumulation [24–26].

The present study applied a transcriptomic approach to investigate the factors that underlie isoflavonoid content variation. The root was chosen as the organ of study; due to the dual importance of isoflavonoids in the root as a) signaling molecules for *nod* gene induction in nitrogen-fixing symbiotic bacteria [27], and b) as phytoalexins and anti-microbial agents warding off pathogen infection [17, 27]. Four soybean cultivars were chosen for this study: two with increased resistance to stem and root rot disease caused by *Phytophthora sojae*, Conrad and AC Colombe; two with susceptibility to *P. sojae*, AC Glengarry and Pagoda (Poysa, personal communication). Multiple sources have cited increased resistance in Conrad soybeans to a broad range of pathogen infection (*Fusarium graminearum* and *P. sojae*) [28, 29].

Here we report that the root isoflavonoid contents of Conrad and AC Colombe are significantly higher than that of AC Glengarry and Pagoda. The variation in isoflavonoid levels in these four soybean cultivars was used as a basis for a transcriptomic approach to find the underlying mechanisms of metabolic variation. Five differentially expressed (DE) genes encoding flavonoid and isoflavonoid metabolic enzymes were found. This list was further refined by quantitative PCR (qPCR) to two genes encoding flavonoid-metabolizing enzymes: flavonoid 3′-hydroxylase (*GmF3′H*) and dihydroflavonol 4-reductase (*GmDFR*). These genes were significantly downregulated in high isoflavonoid cultivars. Transcriptomic downregulation of genes from overlapping phenylpropanoid pathways could divert common substrates, such as naringenin, toward isoflavonoid production. Further DE genes could play a role in transcriptional regulation, metabolism, transport, and plant stress response. The lattermost could be responsible for the favorable resistance traits of Conrad and AC Colombe.

## Methods

### Plant material

Soybean seeds from the cultivars, Conrad, AC Colombe, AC Glengarry and Pagoda, were planted in Pro-Mix BX Mycorrhizae™ soil (Rivière-du-Loup, Canada) in a growth chamber with a 16 h light cycle at 25 °C, and an 8 h dark cycle at 20 °C, with 60-70% relative humidity, with light intensity 100–150 μmol m^−2^s^−1^.

### RNA and isoflavonoid extraction; HPLC analysis

Two week-old soybean roots were harvested, flash-frozen with liquid nitrogen and ground to a fine powder. Three biological replicates were prepared for each of the four cultivars. RNA was extracted from the twelve samples according to the instructions in the RNeasy Mini Kit (Qiagen, Germany). RNA integrity and concentration were recorded using the Agilent 2100 (Bioanalyzer, USA). The same root samples were used for isoflavonoids extraction following the method described previously for seed isoflavonoid extraction and analyzed by high-performance liquid chromatography (HPLC) [7] .

### Quantitative RT-PCR analysis

The same RNA samples used for RNA sequencing were also used in a reverse transcription reaction using the ThermoScript™ RT-PCR System (Invitrogen). Primer sequences for qPCR are listed in Additional file 1: Table S1. All reactions were performed in three technical triplicates, and fold expression was normalized to the reference genes *CONS4* and *CONS6* [30].

### Illumina sequencing, data quality and mapping

Root tissue total RNA from the four cultivars, three replicates per cultivar, was sequenced on an Illumina Hiseq2000 at the DNA Technologies Unit of the Plant Biotechnology Institute (Saskatoon, SK, Canada) using 100 bp paired-end runs. Contaminating adaptor sequences were removed using a custom Perl script, reads subjected to 3′ end trimming (Q ≥ 30) [31]. Reads from each library were mapped against the *Glycine max* transcriptome v2.0, Wm82.a2.v1 [1] using the Burrows-Wheeler Aligner (BWA) [32]. PCR duplicates and reads aligned with a low mapping quality (MQ ≤ 20) were dropped, and uniquely mapped reads per transcript counted using Samtools [32].

### Differential gene expression analysis

Counts for reads mapping to each transcript were imported into the R statistical environment for differential expression analysis with the DESeq package [33]. Transcripts were normalized by counts across biological replicates and cultivars and the bottom 10% of low expressing transcripts dropped. Dispersion estimates were calculated for each gene using DESeq functions to estimate transcript expression variation across replicates for a given cultivar [33]. For the purposes of gene discovery two sets of DE genes were produced based on ‘baseline’ *p* < 0.05 or ‘high’ *p* < 0.001 significance, representing moderate and high stringency. P-values were adjusted using Benjamini-Hochberg multiple-testing correction and all diagnostic and analysis plots generated using DESeq or R functions.

Heatmaps were generated using the R programming package DEseq, and the heatmap.2 function in gplots, accessed from the CRAN library (https://cran.r-project.org/) [34]. Raw gene expression data were normalized for replicate and library differences in read counts and coverage by variance stabilized transformation, using the DESeq package, prior to heatmap generation.

### Functional annotation of differentially expressed transcripts

DE genes were annotated using the soybean transcriptome annotation (Soybean genome assembly version 2.0 in the Phytozome database, Wm82.a2.v1). *Arabidopsis* orthologs were used to associate ‘GO’ annotations (Gene Ontology Database, http://geneontology.org) [35] to soybean genes using the TAIR (The Arabidopsis Information Resource, http://arabidopsis.org) [36].

Over-representation analysis was performed using the ‘PANTHER over-representation test’ (PANTHER version 10, http://pantherdb.org) [37]. Pathway enrichment analysis was carried out using the PhytoMine tool ‘Pathway Enrichment’, (Phytozome version 11, http://phytozome.jgi.doe.gov/phytomine/) [38]. Data from KEGG [39] and PlantCyc (Plant Metabolic Network (PMN), www.plantcyc.org) resources were used to conduct the analysis, with the *Glycine max* database selected as the reference. The Benjamini-Hochberg statistical test was used to adjust *p* values for multiple testing.

## Results

### Stem and root rot resistant soybean cultivars contain high isoflavonoid content in roots

**Fig. 2:**
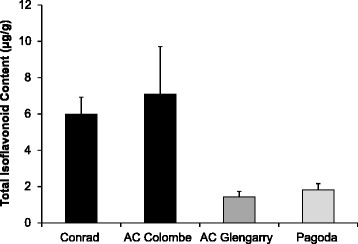
Root isoflavonoid content of four soybean cultivars. Total isoflavonoid content, including the six isoflavonoids, daidzein, genistein, glycitein, daidzin, genistin and glycitin, as measured by HPLC. Isoflavonoids were extracted from the roots of four soybean cultivars: Conrad, AC Colombe, AC Glengarry and Pagoda. Error bars indicate SEM of three independent experiments

Four soybean cultivars were assayed for isoflavonoid content: two with reported resistance, Conrad and AC Colombe, and two with susceptibility to *P. sojae*, AC Glengarry and Pagoda. Total isoflavonoid content includes daidzein, genistein, glycitein and their corresponding glycosides as measured by HPLC analysis. As shown in Fig. 2, Conrad and AC Colombe showed higher total isoflavonoid content in the roots, as compared to AC Glengarry and Pagoda.

### Data quality and coverage of soybean transcriptome in four soybean cultivars

The root transcriptomes of four soybean cultivars were studied, including high, Conrad and AC Colombe, and low, AC Glengarry and Pagoda, root isoflavonoid content. Root mRNA from three replicates for each of the four cultivars was sequenced, using 100 bp paired-end reads. The resulting data were then mapped against the soybean transcriptome v2.0 (Wm82.a2.v1) [1]. Mapping and quality information including total reads per RNA sequencing library, base quality ≥ 30 (Q 30%), mapped reads, uniquely mapped reads and gene coverage (%) are summarized in Table 1. Coverage of the 56,044 protein-coding loci ranged from 62.74 to 65.09%. Biological variation for all the gene models in each cultivar was displayed using dispersion graphs (Additional file 2: Figure S1). Dispersion plots have, overall ‘tight’ dispersion, which is indicative of good quality RNA sequencing libraries for the three biological replicates per cultivar.

**Table 1:** RNA sequencing quality and coverage of soybean transcriptome

	Replicate	Total Reads	Q30 Bases (%)	Mapped Reads	Uniquely Mapped	Gene Coverage (%)
Conrad	1	43642500	92.91	30762485	24744536	64.28
	2	51423168	92.58	36648290	29163761	64.34
	3	41426398	92.67	29497436	23948533	63.96
AC Colombe	1	64237226	92.88	43062343	33956444	65.09
	2	41552636	92.65	29416157	23831591	63.96
	3	50795644	92.41	33359756	27313027	64.41
AC Glengarry	1	33146096	93.52	23522302	19393241	63.34
	2	49338208	93.54	34540611	27773084	64.27
	3	71279056	93.47	49674464	38962472	64.44
Pagoda	1	19820794	93.53	14005991	11582472	62.74
	2	42622034	93.45	30545146	24479762	63.26
	3	40106364	93.67	27711116	22598173	63.64

Coverage of the soybean transcriptome (Wm82.a2.v1) in soybean roots of Conrad, AC Colombe, AC Glengarry and Pagoda biological replicates ranged from 62.74 to 65.09%. Library replicate: the soybean cultivar replicate sequenced; total reads: the total number of sequence reads obtained; Q30 bases (%): the percentage of bases with > 30 quality after 3′ end trimming; mapped reads: the number of reads that mapped to one of the soybean transcript models; uniquely mapped reads: number of mapped reads mapping uniquely to a transcript model with a mapping quality ≥ 20; genes hit: number of gene models with one or more reads mapped to its transcript as a percentage of the total number of gene models (56,044 protein-coding loci)

### Differential expression analyses between high and low root isoflavonoid cultivars

Alignments of RNA sequencing libraries to the soybean transcriptome were imported into R and assessed for differential gene expression using DESeq [33]. The Benjamini-Hochberg adjusted *p*-value distribution for all genes assessed for differential expression between high and low cultivars showed a large number of significantly differentially expressed genes with a uniform dispersion (Additional file 2: Figure S1, Additional file 3: Figure S2, and Additional file 4: Figure S3). To categorize and rank the level of expression variation among these genes, the log *p*-value distribution for all the genes was binned and plotted in terms of significance levels: (*p* < 0.001), (*p* < 1e-15), and (*p* < 1e-55) (Additional file 5: Figure S4). An abundance of gene models being significantly, and highly significantly, differentially expressed in each of the comparisons is representative of the genetic differences between the cultivars.

**Fig. 3:**
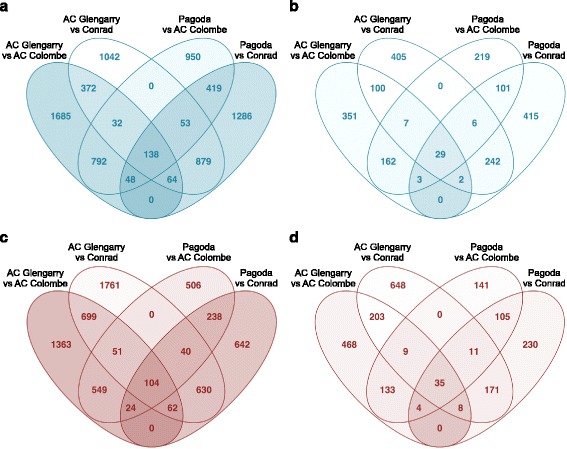
Overlap study of up- and down-regulated genes in high root isoflavonoid cultivars. Venn diagrams depicting the number of (**a, b**) upregulated and (**c, d**) downregulated genes and their overlap in four pair-wise differential expression studies: AC Glengarry with AC Colombe, AC Glengarry with Conrad, Pagoda with AC Colombe, Pagoda with Conrad. Venn diagrams are based on differential expression analysis with ‘baseline’ *p* < 0.05 (**a, c**) or ‘high’ *p* < 0.001 (**b, d**) significance. Therefore, a core of 138 and 104 genes are consistently up- and down-regulated, respectively; the higher *p*-value reduces this list to 29 and 35 highly up- and down-regulated genes, respectively

Four lists were generated from pair-wise differential expression analyses of low against high root isoflavonoid cultivars: AC Glengarry with AC Colombe; AC Glengarry with Conrad; Pagoda with AC Colombe; Pagoda with Conrad. These four lists were filtered to include genes significantly differentially expressed (‘baseline’ *p* < 0.05 or ‘high’ *p* < 0.001) and are compiled in Additional file 6: Table S2, Additional file 7: Table S3, Additional file 8: Table S4, and Additional file 9: Table S5. The lists generated were assessed to find gene models that were consistently up- or down-regulated in all four differential expression analyses. The overlap studies are illustrated in four, four-way Venn diagrams for up- and down-regulation at both baseline and high *p*-values (Fig. 3). The numbers in the ovals represent the number of gene models in the four lists, and their corresponding overlap is also enumerated. The core overlaps of 138 (29) and 104 (35) are the number of genes consistently up- and down-regulated, respectively (with those in parentheses corresponding to *p* < 0.001) (Additional file 6: Table S2, Additional file 7: Table S3, Additional file 8: Table S4, and Additional file 9: Table S5). These lists of genes can be studied for their correlation with isoflavonoid biosynthesis and a putative role in accumulation. The list of up- and down-regulated genes will hereafter be referred to as ‘high isoflavonoid’ and ‘low isoflavonoid’ genes (with ‘v. high/low’ to signify the higher p-value DE genes) (Additional file 6: Table S2, Additional file 7: Table S3, Additional file 8: Table S4, and Additional file 9: Table S5). This does not denote a role in the pathway, but an association with the isoflavonoid content of the cultivars.

Genes that fall outside of this core overlap would include genes up- or down-regulated in one or more of the comparisons but not all four. These genes might represent cultivar-specific differences in the root transcriptome that are not consistently differentially expressed, within the parameters of the study. Heatmaps were generated using raw read counts for the set of 138 ‘high isoflavonoid’ genes, in pair-wise comparisons between low and high root isoflavonoid cultivars (Additional file 10: Figure S5a-d). The heatmaps display the higher expression (*p*-value < 0.05) of the ‘high isoflavonoid’ genes in the cultivars, AC Colombe and Conrad, as compared with AC Glengarry and Pagoda.

### Functional and structural annotation of differentially expressed genes

**Fig. 4:**
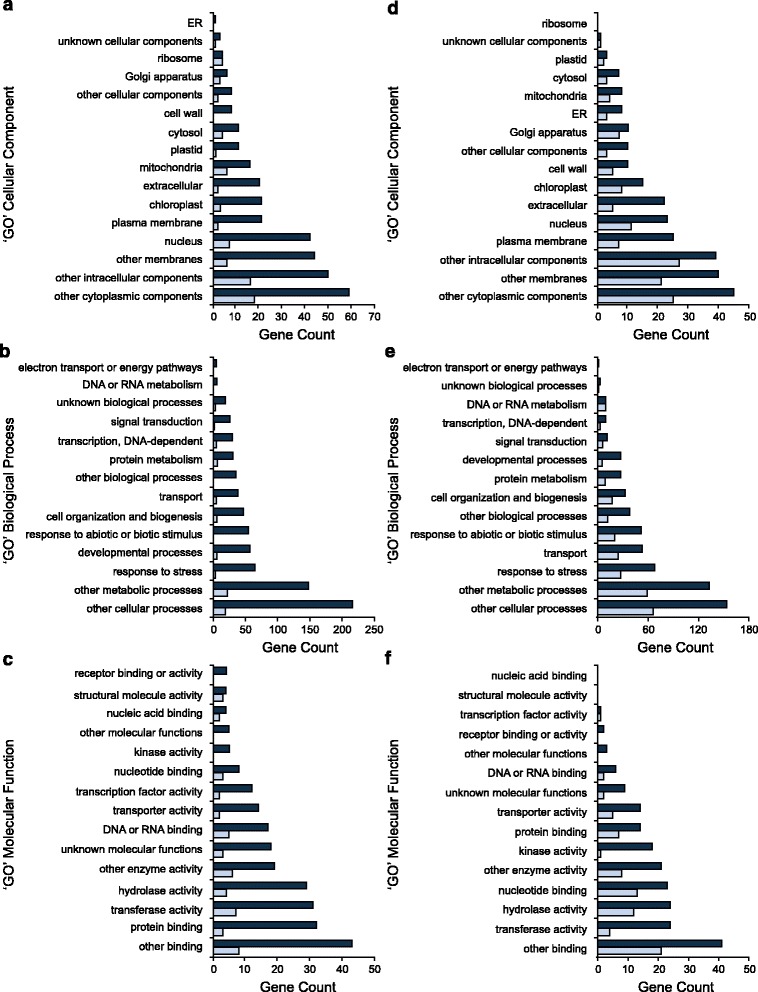
Gene product annotations of upregulated and downregulated genes in high root isoflavonoid cultivars. **a, d** cellular compartment; **b, e** biological processes; **c, f** molecular function annotation, using the Gene Ontology Database annotations of TAIR identifiers that are homologous to differentially expressed soybean genes. **a-c** are upregulated and **d-f** downregulated genes in high root isoflavonoid cultivars. Dark blue and light blue bars denote differentially expressed genes at a *p*-value of <0.05 and <0.001, respectively

*Arabidopsis* genes orthologous to the soybean differentially expressed genes were used to find corresponding ‘GO’ annotations from the TAIR. Figure 4 depicts the putative molecular (Fig. 4c, f) and biological function (Fig. 4b, e), and cellular compartmentalization (Fig. 4a, d) of the protein products for these genes. Figures 4a, b, and c are representative of *p* < 0.05, and Figs. 4d, e, and f are representative of *p* < 0.001 DE genes.

Aside from cytoplasmic proteins, the majority (40) of ‘high isoflavonoid’ gene products were predicted to localize to the nucleus (Fig. 4a), while the plasma membrane is the prominent cellular compartment (24) with ‘low isoflavonoids’ (Fig. 4d). Twelve genes were annotated as transcription factors and three as nucleic acid binding proteins, with the sum of DNA-dependent transcriptional elements coming to seventeen ‘high isoflavonoid’ genes. An Aprataxin-like / bHLH protein was the only ‘low isoflavonoid’ gene product associated with transcription factor activity, putatively involved in DNA metabolism or repair. Five ‘high’ and fifteen ‘low isoflavonoid’ gene products were associated with the ER and golgi, as part of the secretory pathway. Among the ‘high isoflavonoid’ genes, 32 were annotated as being responsive to stress. These genes included member encoding the dirigent family of proteins. Several nucleotide binding disease resistance proteins were identified, containing C-terminal leucine-rich repeat (LRR) domains fused to central nucleotide-binding (NB) domain (NB-LRR proteins) (Table 2). Fifteen ‘high’ and sixteen ‘low isoflavonoid’ gene products upregulated in high isoflavonoid cultivars were annotated for transferase activity. Furthermore, eight ‘high’ and nine ‘low isoflavonoid’ gene products with putative transporter activity were identified.

**Table 2:** Differentially expressed genes that are upregulated (*p* < 0.001) in high isoflavonoid cultivars: DE gene models were inspected manually using annotations from the soybean database (Phytozome), and the ‘GO’ annotations associated with their Arabidopsis homologs (TAIR); they were compiled into families for consideration based on function, and potential involvement in the phenylpropanoid pathway and as underlying factors for isoflavonoid content

	Log_2_ Fold Change						Predicted Cellular Compartment, Biological Processes and Molecular Functions				
Glyma ID	AC Colombe vs.		Conrad vs.		Gene Product Description	*Arabidopsis* Homolog	Cellular Compartment	Metabolism	Transport	Transcriptional Regulation	Stress Response
	AC Glengarry	Pagoda	AC Glengarry	Pagoda							
Glyma.03G065700.1	6.72	6.14	7.13	6.58	GRAS family transcription factor	AT2G37650.1	nucleus			Transcription factor; development	light stress; signal transduction
Glyma.03G070300.1	1.35	1.66	1.53	1.86	serine carboxypeptidase-like 19	AT5G09640.1	apoplast	specialized; sinapoyl-transferase activity; phenylpropanoid role			
Glyma.06G213600.1	1.71	3.85	1.71	3.88	Histone H3 K4-specific methyltransferase SET7/9 family protein	AT4G17080.1	nucleus/cytoplasm	methyl-transferase		histone modification	
Glyma.06G268600.1	2.59	3.06	2.85	3.34	disease resistance protein (TIR-NBS-LRR class)	AT5G17680.1	nucleus/cytoplasm				defense response; signal transduction; cell death
Glyma.06G268700.1	5.06	5.27	5.17	5.38	disease resistance protein (TIR-NBS-LRR class)	AT3G25510.1	nucleus/cytoplasm				defense response; signal transduction; cell death
Glyma.06G308400.1	3.95	4.50	3.35	3.92	alpha/beta-hydrolases superfamily protein	AT5G06570.1	nucleus	carboxylic ester hydrolase activity	amino acid transport		
Glyma.08G087100.1	2.90	2.97	2.54	2.62	thioredoxin-1	AT2G35010.1	cytoplasm	oxidoreductase activity; acts on sulfur group			cell redox homeostasis; symbiosis
Glyma.10G029100.1	4.29	4.61	4.13	4.45	2-oxoglutarate (2OG) and Fe(II)-dependent oxygenase superfamily protein	AT3G63290.1	plasmo-desmata	oxidoreductase activity; pollen development			
Glyma.11G037100.1	2.48	4.11	2.63	4.28	FAD/NAD(P)-binding oxidoreductase family protein	AT2G35660.1	chloroplast/mitochondria	monooxygenase activity			
Glyma.12G188200.1	5.12	7.20	4.60	6.67	histone deacetylase 8	AT1G08460.1	nucleus			histone deacetylase; H3K14 specific; NAD dependent	
Glyma.17G144300.1	1.37	1.41	1.09	1.14	2-oxoglutarate (2OG) and Fe(II)-dependent oxygenase superfamily protein	AT4G16770.1	cytoplasm/peroxisome	oxidoreductase activity; flavonoid biosynthetic process			
Glyma.17G165600.1	1.01	1.13	0.67	0.80	zinc finger protein 7	AT1G24625.1	nucleus			DNA-templated transcriptional regulation;	

The log2 fold changes for the gene models in each of the four pair-wise comparisons between cultivars are detailed in the table

**Fig. 5:**
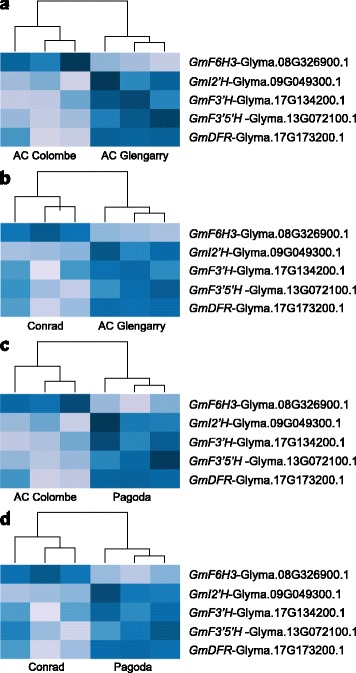
Heatmaps of significantly differentially expressed phenylpropanoid genes. Lists of genes up- or down-regulated consistently were mined for gene models annotated with putative function in the phenylpropanoid pathway. *GmF6H* was upregulated, while *GmI2′H*, *GmF3′H*, *GmF3′5′H*, *GmDFR* were downregulated in high root isoflavonoid cultivars. Data for all three replicates are shown for each soybean cultivar. Panels **a** and **b** show heatmaps of AC Colombe or Conrad with AC Glengarry while panels **c** and **d** show heatmaps of AC Colombe or Conrad with Pagoda

The lists of DE genes were manually inspected for genes putatively involved in specialized metabolism and in particular the isoflavonoid pathway. Several members of oxidoreductase families, including FAD-dependent oxidoreductases (FAD), CYPs, and 2-oxoglutarate (2OG)/Fe(II)-dependent oxygenases (2-ODD) were either up- or down-regulated (Table 2 and 3). Five genes associated directly with the phenylpropanoid pathway (Fig. 1) were identified: flavonoid 6-hydroxylase (*GmF6H*) was upregulated, while isoflavone 2′-hydroxylase (*GmI2′H*), flavonoid 3′-hydroxylase (*GmF3′H*), flavonoid 3′, 5′-hydroxylase (*GmF3′5′H*), and dihydroflavonol 4-reductase (*GmDFR*) were downregulated in high root isoflavonoid cultivars. Heatmaps were also generated to display the differential expression of these five phenylpropanoid genes in the high and low isoflavonoid cultivars (Fig. 5).

**Table 3:** Differentially expressed genes that are downregulated (*p* < 0.001) in high isoflavonoid cultivars: DE gene models were inspected manually using annotations from the soybean database (Phytozome), and the ‘GO’ annotations associated with their Arabidopsis homologs (TAIR); they were compiled into families for consideration based on function, and potential involvement in the phenylpropanoid pathway and as underlying factors for isoflavonoid content

Glyma ID	Log_2_ Fold Change						Predicted Cellular Compartment, Biological Processes and Molecular Functions				
	AC Colombe vs.		Conrad vs.		Gene Product Description	*Arabidopsis* Homolog	Cellular Compartment	Metabolism	Transport	Transcriptional Regulation	Stress Response
	AC Glengarry	Pagoda	AC Glengarry	Pagoda							
Glyma.02G157000.1	−1.31	−1.39	−1.92	−1.98	Inorganic H pyrophosphatase family protein	AT1G15690.2	vacuolar membrane		regulation of apoplastic pH/auxin transport		light-enhanced/repressed by pollen
Glyma.03G066800.1	−0.64	−0.84	−1.08	−1.26	NAD(P)-linked oxidoreductase superfamily protein	AT2G37770.2	chloroplast	NADPH-dependent aldo-keto reductase			upregulated with cold, salt, drought
Glyma.04G151500.1	−1.78	−1.66	−2.67	−2.52	SNF7 family protein	AT5G22950.1	cytosol/endosome membrane		Vacuolar-sorting protein SNF7/protein binding		
Glyma.05G100100.1	−5.09	−5.34	−4.99	−5.22	dsRNA-binding protein	AT2G28380.1	nucleus/cytoplasm			production of miRNAs; silencing	defense response to virus
Glyma.08G070000.1	−2.47	−2.38	−2.17	−2.05	2-oxoglutarate (2OG) and Fe(II)-dependent oxygenase superfamily protein	AT2G36690.1	cytoplasm	oxidoreductase activity; root / hypocotyl			
Glyma.09G279100.1	−0.89	−1.23	−1.36	−1.68	cytochrome P450, family 71, subfamily B, polypeptide 34	AT3G26300.1	integral membrane	CYP71B34; specialized metabolism			
Glyma.14G058600.1	−1.00	−1.09	−1.11	−1.18	2-oxoglutarate (2OG) and Fe(II)-dependent oxygenase superfamily protein	AT3G11150.1	chloroplast	oxidoreductase activity			
Glyma.14G061400.1	−0.93	−0.77	−0.92	−0.76	Nucleotide-diphospho-sugar transferases superfamily protein	AT3G55830.1	integral membrane	Glycosyl-transferase family			cell adhesion in plant development
Glyma.15G031400.1	−8.68	−9.73	−6.69	−7.72	beta glucosidase 15	AT2G44450.1	golgi/cell wall/endomembrane	degradation of flavonol bisglycosides			B-glucosidase activity to abiotic stress
Glyma.17G173200.1	−3.34	−3.15	−2.85	−2.60	dihydroflavonol 4-reductase	AT5G42800.1	ER	dihydroquercetin to leucocyanidin			response to UV-B or sucrose
Glyma.U000400.1	−2.83	−2.47	−5.51	−5.05	APRATAXIN-like/bHLH protein	AT5G01310.1	nucleus	adenylylsulfate sulfohydrolase activity		DNA metabolism and repair	response to DNA damage

The log2 fold changes for the gene models in each of the four pair-wise comparisons between cultivars are detailed in the table

To investigate the significance of the represented GO annotations in the list of genes up- and down-regulated in high isoflavonoid cultivars, PANTHER over-representation test was performed for GO-Slim biological processes, molecular function and cellular component [37] (Additional file 11: Table S6 and Additional file 12: Table S7). Cell structure and morphogenesis elements were overrepresented in the list of upregulated genes. Genes involved in secondary metabolism, specifically those encoding membrane-bound proteins with hydrolase and oxidoreductase activity were overrepresented in the downregulated list. Therefore, a significant number of genes involved in secondary metabolism were downregulated in high isoflavonoid cultivars. Enrichment of specific metabolic pathways in the list of genes up- and down-regulated was analyzed using PhytoMine. The upregulated genes were significantly enriched for phenylalanine metabolism (Additional file 13: Table S8). Downregulated genes were not significantly enriched for any single pathway; however, the classification for flavonoid genes was divided into three categories: flavonoid; flavone and flavonol; stilbenoid, diarylheptanoid and gingerol biosynthesis (Additional file 14: Table S9). The isoflavone-metabolizing I2′H was incorrectly classified under the lattermost category (diarylheptanoid and gingerol biosynthesis), while F3′H was not included in flavonoid biosynthesis.

### Quantitative expression analysis to confirm differential expression candidates

**Fig. 6:**
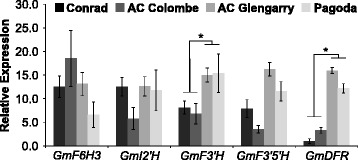
Expression analysis of candidate phenylpropanoid genes in four soybean cultivars. Total RNA was extracted from the roots of soybean cultivars Conrad, AC Colombe, AC Glengarry and Pagoda, and was used in qPCR analysis. Expression of the following genes was analyzed: *GmF6H3*, *GmI2′H*, *GmF3′H*, *GmF3′5′H* and *GmDFR*, using specific primers. Relative expression corresponds to mean gene expression in three biological replicates, with technical triplicates. Error bars indicate SEM. Values were normalized against the reference genes *GmCONS4* and *GmCONS6.* Asterisks (*) indicate significant differences between the samples as determined by Student’s *t* -test

To determine the exact expression levels of differentially expressed candidate genes, and to confirm the predictions of the RNA sequencing results, a qRT-PCR was performed. RNA samples utilized in the making of the sequencing libraries were used for reverse-transcription. Primer sequences were designed to amplify unique regions within target genes: *GmF6H*, *GmI2′H*, *GmF3′H*, *GmF3′5′H* and *GmDFR* (Additional file 1: Table S1). The expression of *GmF3′H* and *GmDFR* were significantly lower in high isoflavonoid cultivars (*p* < 0.05). There was no significant difference in the expression of *GmF6H*, *GmI2′H* and *GmF3′5′H* between the two sets of cultivars (Fig. 6).

## Discussion

The transcriptomic exploration of underlying differences responsible for isoflavonoid content in two sets of soybean cultivars has yielded a large suite of transcripts encoding metabolic enzymes, transcriptional regulators, metabolite transporters and other potentially significant genes. Using RNA sequencing, differential gene expression analysis, and subsequent qPCR analysis of candidate phenylpropanoid metabolic genes, we have identified two DE genes, *GmF3′H* and *GmDFR* that could play a significant role in isoflavonoid accumulation. The enzyme, F3′H, competes with IFS for the branch-point substrate naringenin, while DFR is involved in downstream flavonoid production (Fig. 1). The down-regulation of these putative genes in high isoflavonoid cultivars could describe a mechanism of diverting naringenin, from flavonoid towards isoflavonoid biosynthesis. Functional work would be required to substantiate the link between gene expression and isoflavonoid biosynthesis and/or accumulation.

### Differentially expressed phenylpropanoid genes

Attempts at reconstituting the isoflavonoid pathway in heterologous systems have underlined the importance of competition for flavanone substrates [40–42], particularly naringenin. This has been described as a ‘bottleneck’ [26]; biosynthesis of isoflavonoids increases in mutant backgrounds with reduced or absent flow of substrates into flavonoid metabolism [14, 43, 44]. Another mechanism affecting the level of isoflavone aglycones and their glucoside conjugates is conversion into downstream metabolites such as phytoalexins and signaling molecules [14].

Five differentially expressed genes were identified with phenylpropanoid functional annotations: *GmF6H3*, *GmI2′H*, *GmF3′H*, *GmF3′5′H*, and *GmDFR*. These genes and their corresponding positions in the respective pathways are displayed in Fig. 1. *GmI2′H* (Glyma.09G049300.1) is a putative isoflavonoid pathway gene annotated as a cytochrome P450 (CYP) family 81 (CYP81D3), involved in the NADPH-dependent conversion of isoflavone into 2′-hydroxyisoflavone [45, 46]. Substrates for GmI2′H include the isoflavones, daidzein and genistein, and the isoflavone derivative, formononetin. Therefore, *GmI2′H* encodes a CYP capable of channeling isoflavones into downstream metabolites, and eventually pterocarpan phytoalexins [47]. Downregulation of *GmI2′H* in soybean roots (*p* < 0.05) might lead to decreased flux of isoflavone aglycones into subsequent pathways, thereby accounting for higher levels of daidzein and genistein in Conrad and AC Colombe (Fig. 5). However, differential expression was not confirmed by qPCR analysis (Fig. 6).

Further downregulated phenylpropanoid genes identified by DE analysis include *GmF3′H*, *GmF3′5′H*, and *GmDFR* (Fig. 5)*.* All three are involved in the processing of naringenin to flavonoid metabolism. *GmF3′H* (Glyma.17G134200.1) and *GmF3′5′H* (Glyma.13G072100.1) encode putative CYPs that determine the hydroxylation of the B-ring of flavonoids [48–51]. Together these CYPs, F3′H and F3′5′H, are responsible for the production of dihydroflavonols, which is subsequently converted to leucoanthocyanidin by the action of DFR [52], another flavonoid gene downregulated in high isoflavonoid cultivars (Glyma.17G173200.1). The combined downregulation of these flavonoid pathway genes could play a role in the flux of flavanone substrates (naringenin) into the competing isoflavonoid biosynthesis branch and the increased production of genistein. *GmF3′H* and *GmDFR* were found, by qPCR analysis, to be significantly downregulated in high isoflavonoid cultivars; *GmF3′5′H* was reduced, but not significantly.

The majority of isoflavonoid content in soybean is comprised of daidzein and genistein and their β-glycoside/malonyl derivatives; however, glycitein and its derivatives form the third, and smallest component of this metabolite class. Flavonoid 6-Hydroxylase 3 (*GmF6H3*) (Glyma.08G326900.1) transcript was found at a higher level in high isoflavonoid cultivars (*p* < 0.05) (Fig. 5), and encodes for a CYP enzyme that catalyzes the A-ring hydroxylation of liquiritigenin, synthesizing 6,7,4′-trihydroxyflavanone [53]. GmF6H3 is associated with two other highly similar isoforms GmF6H1 and GmF6H2 of the CYP71D9 family [54]. The intermediate produced in this reaction is further catalyzed by IFS (2-HIS) to produce 2,6,7,4′-trihydroxyflavanone, which undergoes a dehydration (by 2-hydroxyisoflavone dehydratase, 2-HID), and methylation (6-isoflavone-O-methyltransferase 6-IOMT) event to produce glycitein [53]. It has been reported that GmF6H3 is the isoform responsible for glycitein content of soybean seeds, which is almost-exclusively limited to the hypocotyls (embryo-axis), as it was the sole member expressed in the tissue of question [54]. Expression of *GmF6H3* was concurrent with isoflavonoid accumulation in the hypocotyl (25–40 DAP); it was also absent from the seeds of soybean cultivars that are null-mutants for glycitein accumulation. Strikingly, GmF6H3 unlike the other members of the GmF6H family lacks the characteristic N-terminal hydrophobic transmembrane domain that anchors CYP enzymes into the ER [54]. *GmF6H3* gene expression, as analyzed by qPCR (Fig. 6), did not correlate with high isoflavonoid accumulation (Fig. 2). The discrepancy between qPCR and differential expression analysis results for *GmF6H3, GmI2′H* and *GmF3′5′H* could indicate that the DE results were not accurate to the same level as qPCR analysis, as these genes fell in the ‘baseline’ *p*-value score (*p* < 0.05) (Fig. 6). Alternatively, the gene expression and function of a different isoform of the *F6H* or *I2′H* gene families could correlate with high isoflavonoid content, as assigning reads to highly similar genetic regions is problematic.

### Enzymatic candidates for a role in specialized metabolism

Table 2 and Table 3 are a compilation of manually selected candidates from the high stringency (*p* < 0.001) DE analysis that might be associated with the isoflavonoid content of the four cultivars in question. In addition to *GmDFR* that is significantly downregulated, there are several CYPs, 2-ODDs and FAD/NADP-dependent oxidoreductases in the list that could be top candidates for involvement in specialized metabolism. Overrepresentation and pathway enrichment statistics (Additional file 11: Tables S6, Additional file 12: Tables S7, Additional file 13: Tables S8, and Additional file 14: Tables S9) show that genes involved in secondary metabolism, particularly those encoding hydrolase and oxidoreductase activity are significantly enriched in the ‘low isoflavonoid’ gene list. Membrane integral proteins are also overrepresented within this list, which could impact the formation of isoflavonoid or flavonoid multi-enzyme complexes or ‘metabolons’ [8, 55–57].

2-ODDs are a very versatile family of dioxygenases that are superior to CYPs in the range of reactions they catalyze, from hydroxylations to ring fragmentation [58]. They are ubiquitously distributed throughout nature, and have very important roles in core and specialized metabolic pathways [59], including histone demethylation [60], and the biosynthesis of gibberellic acid [61], flavonoids [62], benzylisoquinoline alkaloids [63], and glucosinalates [64], to name a few. It is important to note that flavanone 3β-hydroxylase (F3H) is a 2-ODD, converting a 2*S*-flavanone to dihydroflavonols by a C-3 hydroxylation [62, 65]. This is an enzyme that competes with isoflavonoid biosynthesis, and downregulation of a corresponding gene could underlie increased isoflavonoid content. There are several reactions in the flavonoid and isoflavonoid pathways that have yet to be elucidated and Table 2 and Table 3 could provide a starting point towards gene discovery. Among them there are four putative 2-ODD genes, two that are upregulated: Glyma.10G029100.1.p and Glyma.17G144300.1.p, and two downregulated members: Glyma.08G070000.1.p and Glyma.14G058600.1.p.

Other metabolic enzymes represented in the list of high stringency DE genes include a downregulated homolog of CYP71B34 (Glyma.09G279100.1.p), and several FAD/NAD(P)-binding oxidoreductases (upregulated: Glyma.11G037100.1.p; downregulated: Glyma.03G066800.1.p) (Tables 2 and 3). The latter group has been less present within the current literature on phenylpropanoid pathway; however an NADPH/FAD-dependent enzyme was shown to be responsible in the additional hydroxylation of positions 6 and/or 8 of the flavonol A-ring [66] This provides a precedence for FAD/NAD(P)-linked oxidoreductases being involved in the content and composition of phenylpropanoids, and should be further researched.

### Stress response in high isoflavonoid cultivars

Isoflavonoid biosynthesis is inducible by several abiotic and biotic stimuli. Therefore, it would be interesting to further investigate the 27 ‘high isoflavonoid’ genes that are annotated as being responsive to stress (Additional file 15: Table S10). Subsets of these genes are annotated as being involved in disease resistance such as, dirigent proteins, which dictate the stereochemistry of other proteins [67], and several nucleotide binding disease resistance proteins. The latter included NB-LRR proteins that are associated with *R* gene function [68, 69]. Several of these genes were annotated as being root hair specific. As root hairs are often the site for pathogen entry, the convergence of tissue-specialization and function could be indicative of a role for these genes in inhibiting pathogen infection or colonization.

As mentioned earlier, Conrad and AC Colombe are also favored for their increased resistance to *P. sojae* (Poysa, personal communication) and other soybean pathogens [28, 29]. Therefore, the differential expression of genes involved in stress response, localized to the root hairs could be suggestive of a mechanism underlying the improved resistance to pathogen infection.

### Transcriptional regulation: transcription factors and chromatin regulators

Subcellular localization of the ‘high isoflavonoid’ genes to the nucleus highlights the potential role of the cognate proteins in transcriptional regulation. The list of 40 nuclear-localized genes includes 12 transcriptional regulators (Additional file 16: Table S11), including members of the bHLH superfamily of proteins and MYB transcription factors. Physical interaction and regulatory synergy has been shown between these family of proteins in regulating gene expression [70]. Combinatorial plant gene regulation might be a factor in the coordinated redirection of flux in specialized metabolism, leading to isoflavonoid content variation in soybean cultivars. A GRAS family transcription factor (Glyma.03G065700.1.p) was significantly upregulated (*p* < 0.001) in high isoflavonoid cultivars (Table 2). Based on homology to *Arabidopsis* transcription factors it is putatively denoted for stress transduction. As isoflavonoid production is closely related to stress response, this transcription factor could be an important candidate for further study.

Another function for nuclear-localized proteins could be the regulation of chromatin structure, which thereby effects broad transcriptomic changes. Two genes upregulated (*p* < 0.001) and annotated for such function were identified: a SET7/9 family, histone H3K4-specific methyltransferase protein and a histone deacetylase 8 (*GmHDA8*) family protein (Table 2). The former, a histone-modifier protein, cannot be immediately associated with activation or repression of transcriptional regulation, as methylation of H3K4 would have to be regarded in the larger landscape and context of methylations [71].

*GmHDA8*, on the other hand, could be associated with the downregulation of certain genes, explaining the transcriptomic changes between cultivars. Deacetylation conferred by elements such as GmHDA8 can increase DNA-histone affinity and condense the overall structure, rendering it inaccessible to transcription machinery [72]. Hypoacyetylated chromatin is silent, or has reduced gene expression. Therefore, GmHDA8 might be a histone-modifying element responsible for some of the overall changes in the root transcriptome that coincide between the high isoflavonoid cultivars, Conrad and AC Colombe.

### Vacuolar sequestration: a putative regulatory mechanism for isoflavonoid accumulation

Isoflavone aglycones produced by an enzyme complex tethered to the ER, are conjugated by transferases, allowing their appropriate channeling into the vacuole [14]. From this storage point, isoflavonoids can be sequestered outside of the metabolic flux and released upon demand for stress response or as signaling molecules for symbiosis with nitrogen-fixing rhizobia [14]. Interestingly, there were 15 up- and 16 down-regulated genes annotated for transferase activity; 8 up- and 9 down-regulated genes with putative transporter activity; the suite of transferases and transporters being simultaneously regulated could denote a preference towards the conjugation and storage of a certain class of metabolites (Additional file 6: Table S2, and Additional file 7: Table S3). However, there is still ambiguity over the specificity of vacuolar transporters involved in the channeling of specialized metabolites, and broad substrate acceptance within classes of compounds has been reported [73–75].

Interestingly, one of the candidate upregulated genes identified in the DE analysis of soybean cultivars, was an uncharacterized member of the MATE family (Glyma.10G267800), and hereafter referred to as *GmMATE10*. MATE transporters are important families of proteins in the glycosylation and the subsequent transport of isoflavones [76]. *GmMATE10* was consistently upregulated in high isoflavonoid cultivars, suggesting a possible role in the sequestration of isoflavonoids. The predicted localization of this protein was to the secretory pathway, based on the presence of signal peptide. This could imply localization to the ER, golgi bodies or the vacuole, with the last option being highly probable, given its functional annotation as a MATE transporter. The deduced amino acid sequence of this gene had an amino acid sequence similarity of 32.47% with the TT12 homolog in *Arabidopsis,* based on Clustal W [77], the latter being a known proanthocyanidin vacuolar transporter [76].

MATE transporters in *Medicago truncatula*, MATE1 and MATE2, are involved in transport of phenylpropanoids. MATE1 is a tonoplast epicatechin 3′-*O-*glucoside (E3′G) transporter [78]. MATE2 is a flavonoid transporter involved in vacuolar sequestration of anthocyanins and other flavonoids in flowers and leaves. MATE2 transporter prefers malonylated flavonoid glucosides and is co-expressed with three genes encoding malonyltransferases [76]. The discovery of an isoflavonoid-specific MATE transporter, in turn, could be used as a tool to investigate co-expressed transferases, depicting a more complete image of the conjugation and transport of isoflavonoids.

Furthermore, up-regulation of *GmMATE10* could indicate a mechanism for the vacuolar transport and sequestration of isoflavonoids and/or precursors, in competition with parallel pathways for metabolic flux.

## Conclusions

The coordinated regulation of genes encoding enzymes, transporters, transcription factors and other molecular elements could lead to the increase of root isoflavonoids in Conrad and AC Colombe. These transcriptomic elements can help overcome bottlenecks in isoflavonoid production for soybean cultivars, legumes, and heterologous systems. Genes annotated for disease and stress response could be important in conferring soybean resistance to *P. sojae* infection in Conrad and AC Colombe. Functional genomics should be employed to characterize the role of such transcriptomic elements in isoflavonoid production and disease resistance.

## Additional files

Additional file 1:
**Table S1.** Sequence of oligonucleotides used for qPCR of soybean genes in high and low root isoflavonoid cultivars to confirm differential expression analysis from RNAseq. (DOCX 16 kb)
Additional file 2:
**Figure S1.** Estimated intra-cultivar dispersion for all genes. Dispersion values plotted as a function of average normalized expression for each gene model. Biological variation displayed for **(a)** Conrad **(b)** AC Colombe **(c)** AC Glengarry and **(d)** Pagoda in three biological replicates. (PDF 298 kb)
Additional file 3:
**Figure S2.** M-A plot of log2 fold change as a function of average expression of gene. Significantly differentially expressed genes (FDR ≤ 0.001) are represented in red. Plots displayed for pair-wise comparisons **(a)** AC Glengarry with AC Colombe **(b)** AC Glengarry with Conrad **(c)** Pagoda with AC Colombe **(d)** Pagoda with Conrad. (PDF 493 kb)
Additional file 4:
**Figure S3.** Gene frequency as a function of Benjamini-Hochberg adjusted p-values. Histogram of p-values for all genes assessed for significant differential expression between two cultivars **(a)** AC Glengarry with AC Colombe **(b)** AC Glengarry with Conrad **(c)** Pagoda with AC Colombe **(d)** Pagoda with Conrad. (PDF 326 kb)
Additional file 5:
**Figure S4.** Genes ranked by total read count against –log10 of the p-value. Significance scores divided into three levels: (*p* < 0.001), (*p* < 1e-15), and (*p* < 1e-55). Average gene counts divided into percentiles. Four plots representing significance of differential expression between cultivars **(a)** AC Glengarry with AC Colombe **(b)** AC Glengarry with Conrad **(c)** Pagoda with AC Colombe **(d)** Pagoda with Conrad. (PDF 486 kb)
Additional file 6:
**Table S2.** List of genes upregulated in high (Conrad and AC Colombe) as compared with low (AC Glengarry and Pagoda) root isoflavonoid content cultivars. Differentially expressed genes (*p* < 0.05) in the four comparisons between high and low cultivars were analyzed for overlap (Fig. 2), generating a set of 138 candidates upregulated consistently in high root isoflavonoid cultivars. (DOCX 27 kb)
Additional file 7:
**Table S3.** List of genes downregulated in high (Conrad and AC Colombe) as compared with low (AC Glengarry and Pagoda) root isoflavonoid content cultivars. Differentially expressed genes (*p* < 0.05) in the four comparisons between high and low cultivars were analyzed for overlap (Fig. 2), generating a set of 104 candidates downregulated consistently in high root isoflavonoid cultivars. (DOCX 22 kb)
Additional file 8:
**Table S4.** List of genes upregulated in high (Conrad and AC Colombe) as compared with low (AC Glengarry and Pagoda) root isoflavonoid content cultivars. Highly differentially expressed genes (*p* < 0.001) in the four comparisons between high and low cultivars were analyzed for overlap (Fig. 2), generating a set of 29 candidates upregulated consistently in high root isoflavonoid cultivars. (DOCX 16 kb)
Additional file 9:
**Table S5.** List of genes downregulated in high (Conrad and AC Colombe) as compared with low (AC Glengarry and Pagoda) root isoflavonoid content cultivars. List of genes downregulated in high (Conrad and AC Colombe) as compared with low (AC Glengarry and Pagoda) root isoflavonoid content cultivars. Highly differentially expressed genes (*p* < 0.001) in the four comparisons between high and low cultivars were analyzed for overlap (Fig. 2), generating a set of 35 candidates downregulated consistently in high root isoflavonoid cultivars. These genes were annotated using the soybean database and have been compiled below. (DOCX 17 kb)
Additional file 10:
**Figure S5.** Heatmaps of genes upregulated in high root isoflavonoid cultivars. Read counts for gene models were normalized across cultivars. Four heatmaps representing genes upregulated in high isoflavonoid cultivars as compared with low root isoflavonoid cultivars were generated (three columns per cultivar representing biological replicates): **(a)** AC Glengarry with AC Colombe **(b)** AC Glengarry with Conrad **(c)** Pagoda with AC Colombe **(d)** Pagoda with Conrad. (PDF 417 kb)
Additional file 11:
**Table S6.** Genes upregulated in ‘high isoflavonoid’ cultivars (138 genes) were analyzed for overrepresentation of PANTHER GO-Slim classifications for: biological process, molecular function and cellular component. (DOCX 18 kb)
Additional file 12:
**Table S7.** Genes downregulated in ‘high isoflavonoid’ cultivars (104 genes) were analyzed for overrepresentation of PANTHER GO-Slim classifications for: biological process, molecular function and cellular component. (DOCX 21 kb)
Additional file 13:
**Table S8.** Genes upregulated in ‘high isoflavonoid’ cultivars (138 genes) were analyzed for pathway enrichment. (DOCX 19 kb)
Additional file 14:
**Table S9.** Genes downregulated in ‘high isoflavonoid’ cultivars (104 genes) were analyzed for pathway enrichment. (DOCX 20 kb)
Additional file 15:
**Table S10.** Genes annotated with disease or stress response and upregulated in ‘high isoflavonoid’ cultivars. (DOCX 18 kb)
Additional file 16:
**Table S11.** Genes annotated for transcriptional regulation and upregulated in ‘high isoflavonoid’ cultivars. (DOCX 16 kb)

## Abbreviations

CHI: Chalcone isomerase
CHR: Chalcone reductase
CHS: Chalcone synthase
CYP: Cytochrome P450
DE: Differentially expressed
GmDFR: Soybean dihydroflavonol 4-reductase
GmF3′H: Soybean flavonoid 3′-hydroxylse
GO: Gene ontology
