# Supplementary material for: Transcriptomic evidence for the control of soybean root isoflavonoid content by regulation of overlapping phenylpropanoid pathways
Source: BMC Genomics. 2017 Jan 11;18:70. doi: 10.1186/s12864-016-3463-y (PMC5225596; doi:10.1186/s12864-016-3463-y)
Supplement: Additional file 1: — Table S1. Sequence of oligonucleotides used for qPCR of soybean genes in high and low root isoflavonoid cultivars to confirm differential expression analysis from RNAseq. (DOCX 16 kb) [file 12864_2016_3463_MOESM1_ESM.docx]

**Table S1** Sequence of oligonucleotides used for qPCR of soybean genes in high and low root isoflavonoid cultivars to confirm differential expression analysis from RNAseq.

| **Gene** | **Primer Name** | **Sequence** | **Amplicon Size (bp)** |
| --- | --- | --- | --- |
| ***GmF6H3*** | F6H-F | 5'- GCC CAA TAA TTG ATG ACG AAG GTT C -3' | 228 |
|  | F6H-R | 5'- GTT CCC TAT GAT AGG TAG CTT CCA TG -3' |  |
| ***GmI2’H*** | I2H-F | 5'- GTG CAA CAA TCA AGT ACT CAT CAG C -3' | 188 |
|  | I2H-R | 5'- GGT GAA TAG GTT GTT CGA GGA GG -3' |  |
| ***GmF3’H*** | F3H-F | 5'- CCT GTA CCT CAT CCA CCC ATC TTC -3' | 165 |
|  | F3H-R | 5'- GGA AGT AGA GGC ATG AGT ACC AAG C -3' |  |
| ***GmF3’5’H*** | F3'5'H-F-MD1 | 5’-TTG ATC ACT CGT CTC TCC ATT C-3’ | 187 |
|  | F3'5'H-R-MD1 | 5’-CCA TGT TGT TAG TGC CCA TTT T-3’ |  |
| ***GmDFR*** | DFR-F-MD1 | 5’-CAA GTC TCA TGG TTG GAT CAC TA-3’ | 133 |
|  | DFR-RN | 5’-GAG AAC ACT GGG AAC GAC ACG-3’ |  |
