# Supplementary figures and images for: Transcriptomic evidence for the control of soybean root isoflavonoid content by regulation of overlapping phenylpropanoid pathways
Source: BMC Genomics. 2017 Jan 11;18:70. doi: 10.1186/s12864-016-3463-y (PMC5225596; doi:10.1186/s12864-016-3463-y)

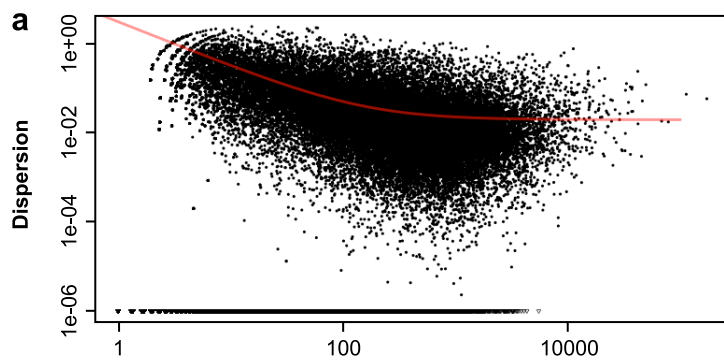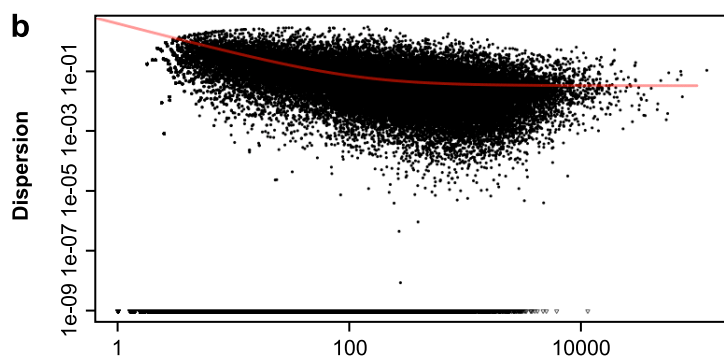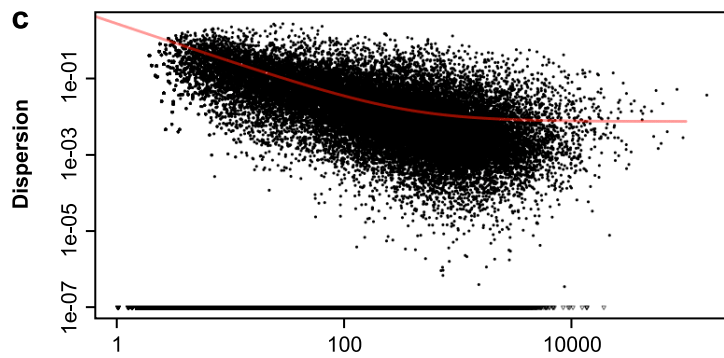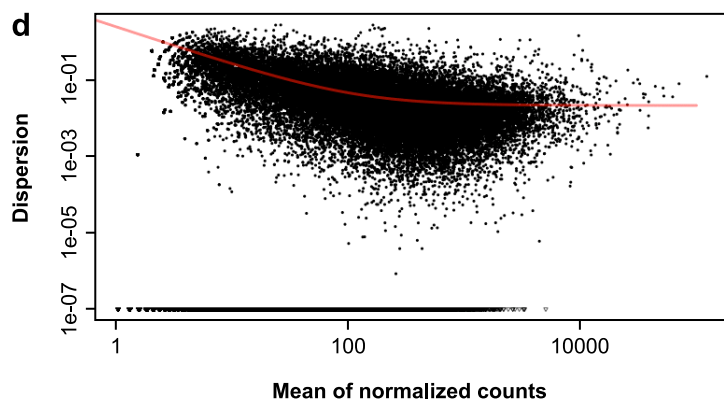

Supplement: Additional file 2: — Figure S1. Estimated intra-cultivar dispersion for all genes. Dispersion values plotted as a function of average normalized expression for each gene model. Biological variation displayed for (a) Conrad (b) AC Colombe (c) AC Glengarry and (d) Pagoda in three biological replicates. (PDF 298 kb) [file 12864_2016_3463_MOESM2_ESM.pdf]

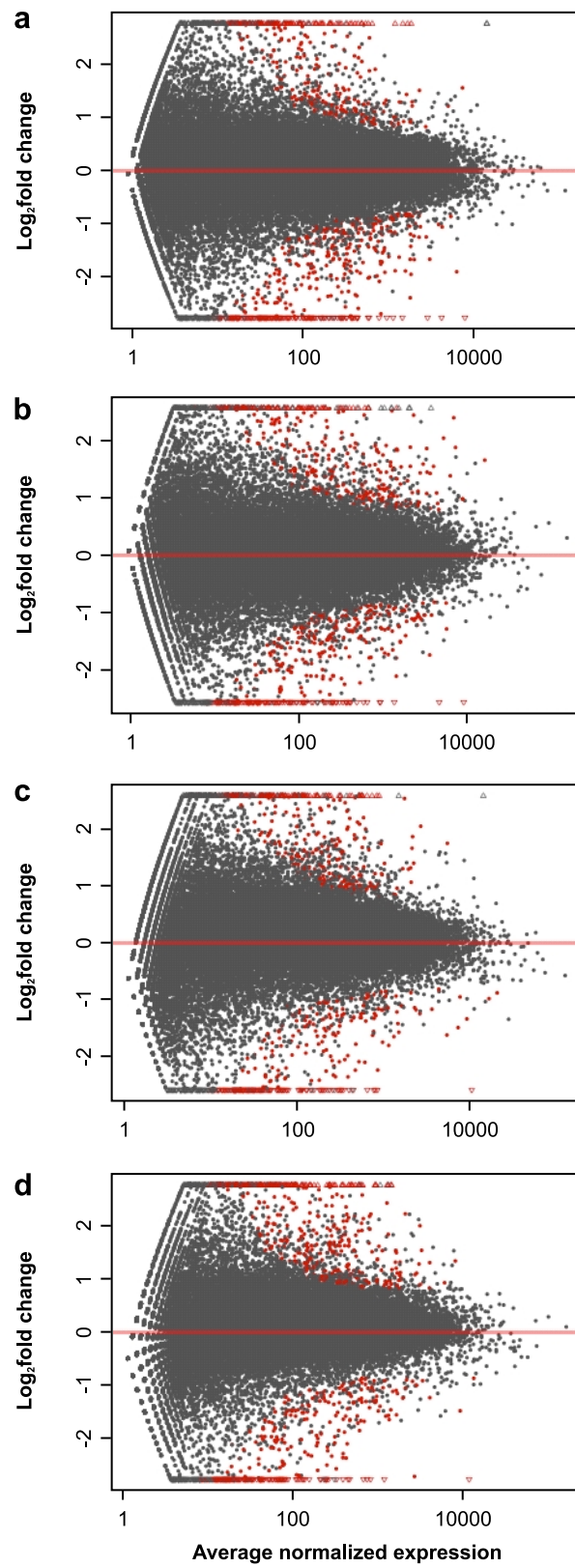

Supplement: Additional file 3: — Figure S2. M-A plot of log2 fold change as a function of average expression of gene. Significantly differentially expressed genes (FDR ≤ 0.001) are represented in red. Plots displayed for pair-wise comparisons (a) AC Glengarry with AC Colombe (b) AC Glengarry with Conrad (c) Pagoda with AC Colombe (d) Pagoda with Conrad. (PDF 493 kb) [file 12864_2016_3463_MOESM3_ESM.pdf]

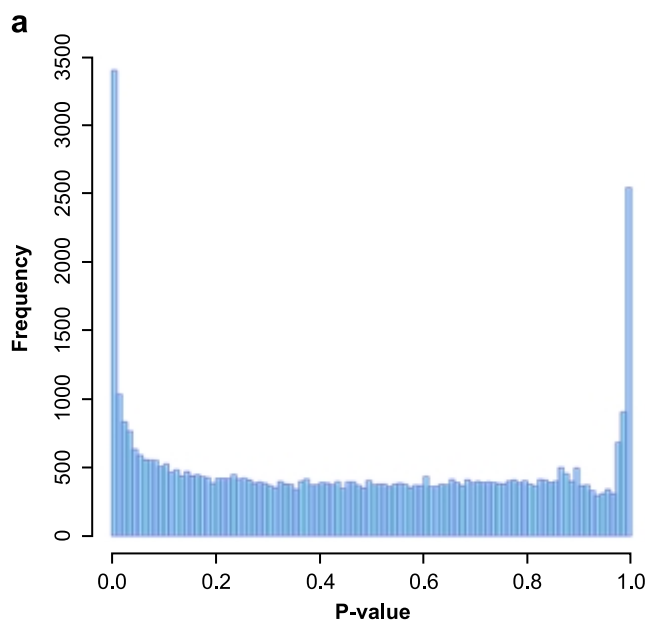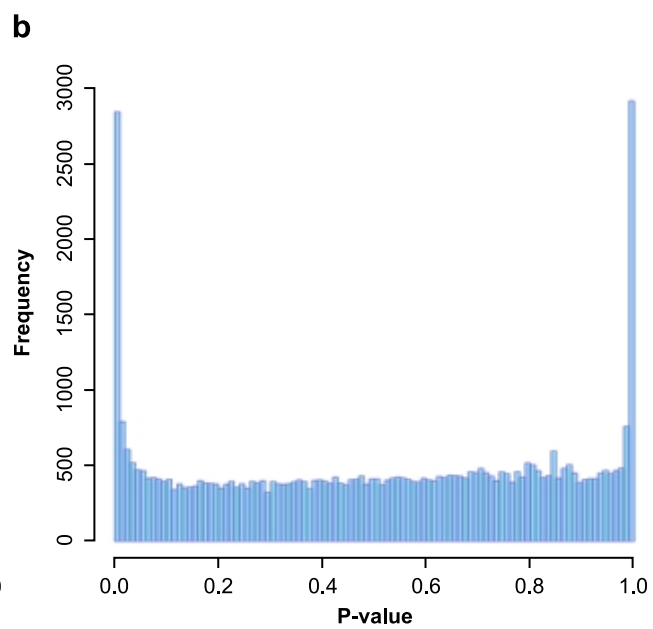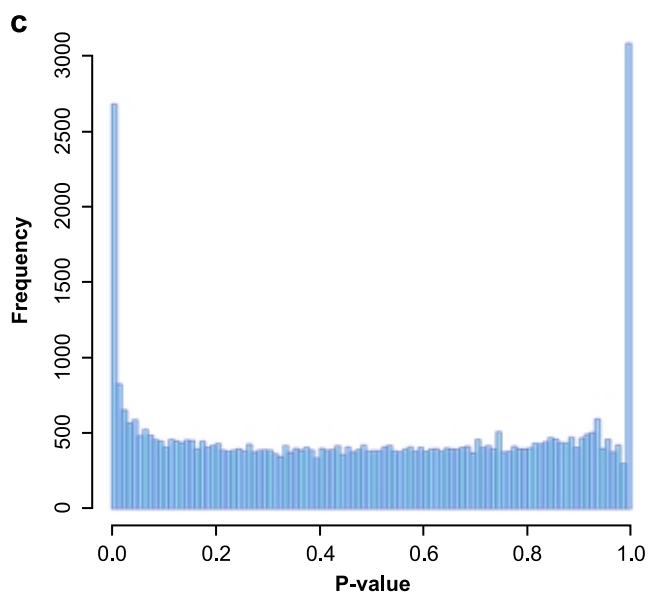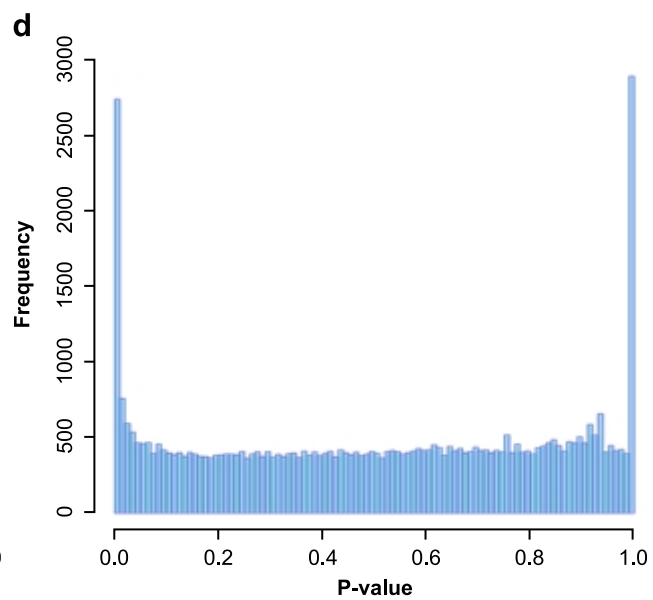

Supplement: Additional file 4: — Figure S3. Gene frequency as a function of Benjamini-Hochberg adjusted p-values. Histogram of p-values for all genes assessed for significant differential expression between two cultivars (a) AC Glengarry with AC Colombe (b) AC Glengarry with Conrad (c) Pagoda with AC Colombe (d) Pagoda with Conrad. (PDF 326 kb) [file 12864_2016_3463_MOESM4_ESM.pdf]

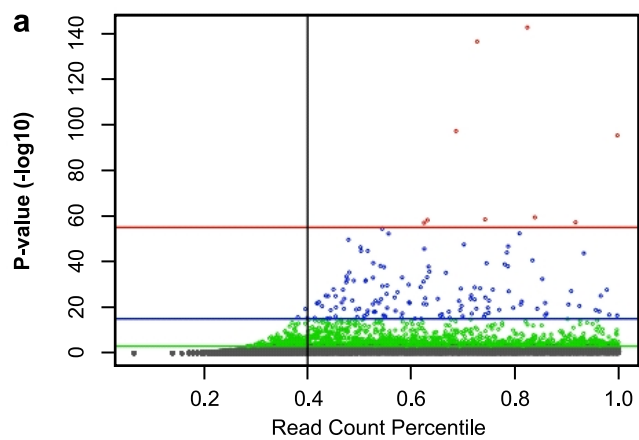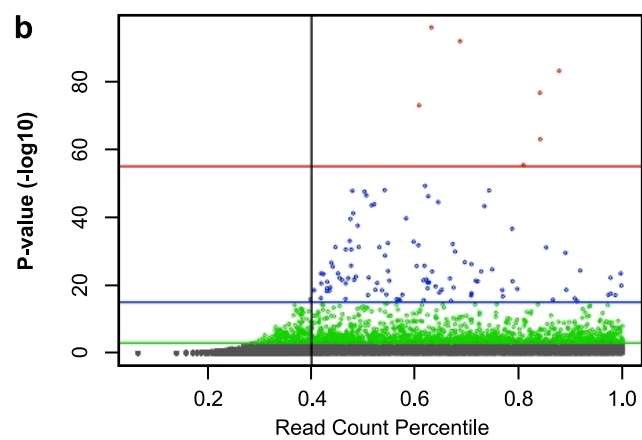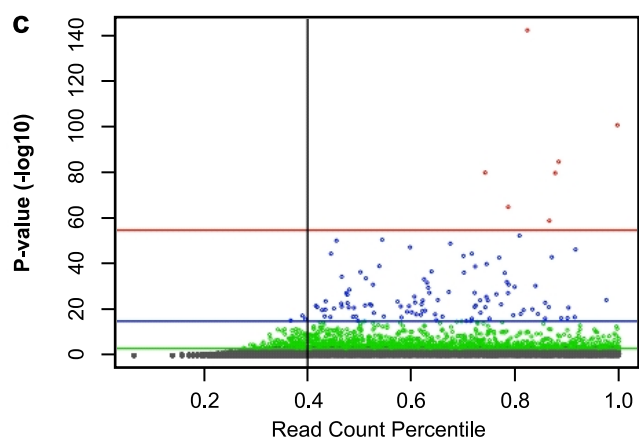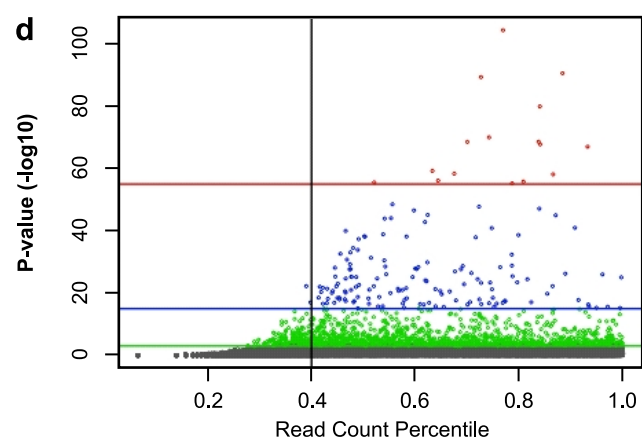

Supplement: Additional file 5: — Figure S4. Genes ranked by total read count against –log10 of the p-value. Significance scores divided into three levels: (p < 0.001), (p < 1e-15), and (p < 1e-55). Average gene counts divided into percentiles. Four plots representing significance of differential expression between cultivars (a) AC Glengarry with AC Colombe (b) AC Glengarry with Conrad (c) Pagoda with AC Colombe (d) Pagoda with Conrad. (PDF 486 kb) [file 12864_2016_3463_MOESM5_ESM.pdf]

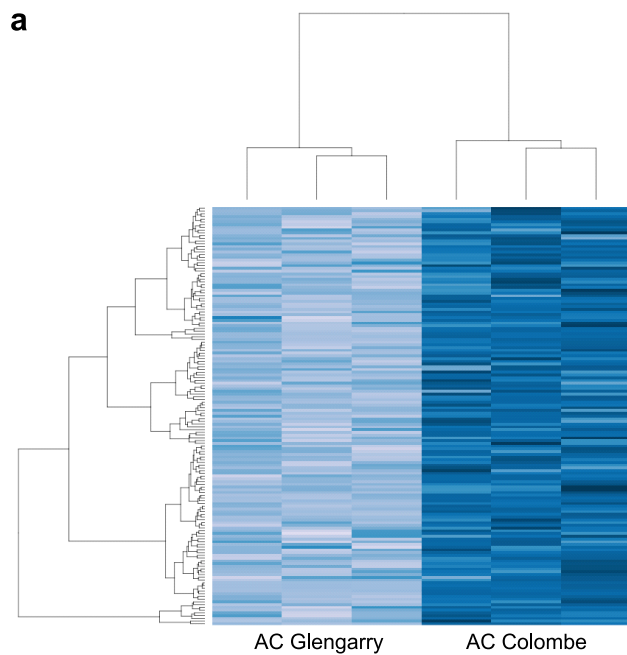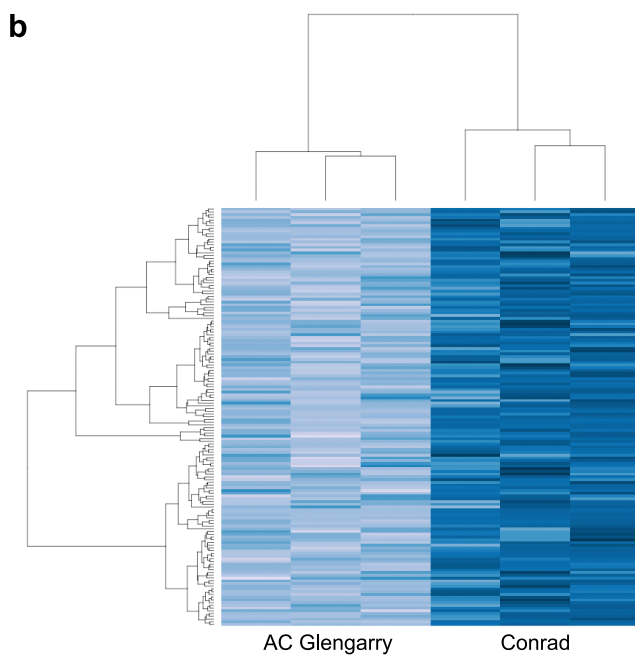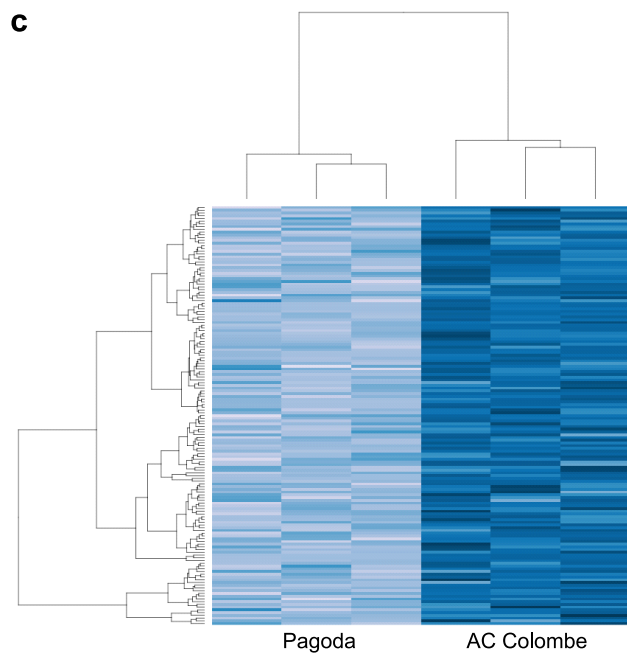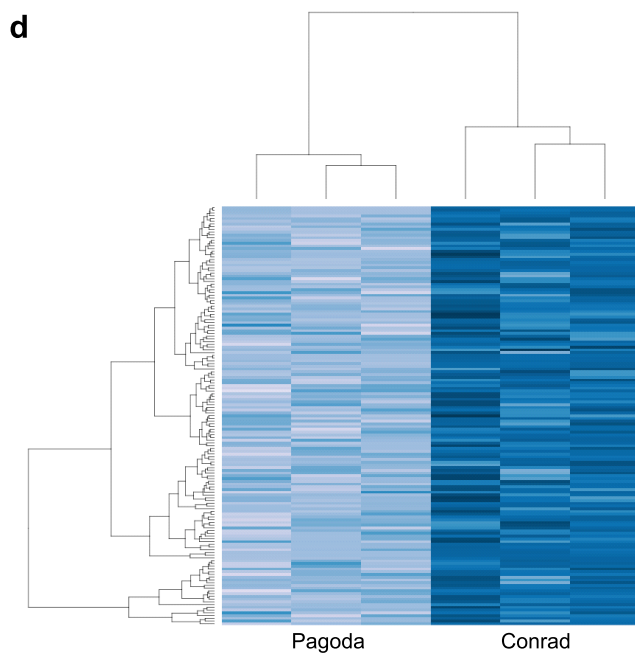

Supplement: Additional file 10: — Figure S5. Heatmaps of genes upregulated in high root isoflavonoid cultivars. Read counts for gene models were normalized across cultivars. Four heatmaps representing genes upregulated in high isoflavonoid cultivars as compared with low root isoflavonoid cultivars were generated (three columns per cultivar representing biological replicates): (a) AC Glengarry with AC Colombe (b) AC Glengarry with Conrad (c) Pagoda with AC Colombe (d) Pagoda with Conrad. (PDF 417 kb) [file 12864_2016_3463_MOESM10_ESM.pdf]
