# Supplementary material for: Transcriptomic evidence for the control of soybean root isoflavonoid content by regulation of overlapping phenylpropanoid pathways
Source: BMC Genomics. 2017 Jan 11;18:70. doi: 10.1186/s12864-016-3463-y (PMC5225596; doi:10.1186/s12864-016-3463-y)
Supplement: Additional file 6: — Table S2. List of genes upregulated in high (Conrad and AC Colombe) as compared with low (AC Glengarry and Pagoda) root isoflavonoid content cultivars. Differentially expressed genes (p < 0.05) in the four comparisons between high and low cultivars were analyzed for overlap (Fig. 2), generating a set of 138 candidates upregulated consistently in high root isoflavonoid cultivars. (DOCX 27 kb) [file 12864_2016_3463_MOESM6_ESM.docx]

**Table S2** List of genes upregulated in high (Conrad and AC Colombe) as compared with low (AC Glengarry and Pagoda) root isoflavonoid content cultivars. Differentially expressed genes (p<0.05) in the four comparisons between high and low cultivars were analyzed for overlap (Fig. 2), generating a set of 138 candidates upregulated consistently in high root isoflavonoid cultivars. These genes were annotated using the soybean database and have been compiled below:

| **Glyma identifier** | **Annotation (domain and motif description)** |
| --- | --- |
| Glyma.01G000800.1 | RNA recognition motif |
| Glyma.01G118900.1 | Leucine-rich repeat transmembrane protein kinase |
| Glyma.01G122300.1 | Glycine-rich protein |
| Glyma.01G124000.1 | Protein of unknown function (DUF688) |
| Glyma.01G127400.1 | Disease resistance-responsive (dirigent-like protein) family protein |
| Glyma.01G243400.1 | Uncharacterized protein |
| Glyma.02G008200.1 | RAD-like 6 |
| Glyma.02G102400.1 | Thioredoxin superfamily protein |
| Glyma.02G108100.1 | Galactosyltransferase1 |
| Glyma.02G139500.1 | Formin homology 1 |
| Glyma.02G264800.1 | RING/U-box superfamily protein |
| Glyma.03G047000.1 | LRR and NB-ARC domains-containing disease resistance protein |
| Glyma.03G061200.1 | Plant protein of unknown function (DUF247) |
| Glyma.03G061300.1 | Uncharacterized protein |
| Glyma.03G065700.1 | GRAS family transcription factor |
| Glyma.03G067100.1 | Uncharacterized protein family (UPF0497) |
| Glyma.03G068900.1 | AtGCP3 interacting protein 1 |
| Glyma.03G069100.1 | RmlC-like cupins superfamily protein |
| Glyma.03G070300.1 | Serine carboxypeptidase-like 19 |
| Glyma.03G125300.1 | AtGCP3 interacting protein 1 |
| Glyma.03G248300.1 | MuDR family transposase |
| Glyma.04G110500.1 | Uncharacterized protein |
| Glyma.04G151000.1 | Homeodomain-like superfamily protein |
| Glyma.04G226800.1 | Calcium-binding endonuclease/exonuclease/phosphatase family |
| Glyma.05G100900.1 | Zinc finger protein 7 |
| Glyma.05G185900.1 | Uncharacterized conserved protein UCP009193 |
| Glyma.06G014200.1 | Uncharacterized protein |
| Glyma.06G027000.1 | Salt tolerance homolog 2 |
| Glyma.06G134900.1 | Heat shock protein 21 |
| Glyma.06G138700.1 | Ribosomal protein L6 family |
| Glyma.06G178400.1 | Copper amine oxidase family protein |
| Glyma.06G213600.1 | Histone H3 K4-specific methyltransferase SET7/9 family protein |
| Glyma.06G268600.1 | Disease resistance protein (TIR-NBS-LRR class), putative |
| Glyma.06G268700.1 | Disease resistance protein (TIR-NBS-LRR class), putative |
| Glyma.06G274200.1 | Ribosomal L5P family protein |
| Glyma.06G277600.1 | ATP synthase protein I -related |
| Glyma.06G308400.1 | Alpha/beta-Hydrolases superfamily protein |
| Glyma.06G313600.1 | Zinc induced facilitator-like 1 |
| Glyma.06G317900.1 | PLAC8 family protein |
| Glyma.07G034600.1 | CASC3/Barentsz eIF4AIII binding |
| Glyma.07G141600.1 | Ctr copper transporter family |
| Glyma.07G143500.1 | Receptor like protein 13 |
| Glyma.07G181300.1 | Hydroxyproline-rich glycoprotein family protein |
| Glyma.07G181600.1 | K-box region and MADS-box transcription factor family protein |
| Glyma.08G022300.1 | Glycosyl hydrolase 9C2 |
| Glyma.08G024800.1 | RING/U-box superfamily protein |
| Glyma.08G038000.1 | Uncharacterized protein |
| Glyma.08G062800.1 | BAK1-interacting receptor-like kinase 1 |
| Glyma.08G074100.1 | Bifunctional inhibitor/lipid-transfer protein/seed storage 2S albumin superfamily protein |
| Glyma.08G087100.1 | Thioredoxin O1 |
| Glyma.08G194900.1 | Pyridoxal phosphate phosphatase-related protein |
| Glyma.08G272100.1 | VQ motif-containing protein |
| Glyma.08G326700.1 | Plant transposase (Ptta/En/Spm family) |
| Glyma.08G326900.1 | Cytochrome P450, family 71, subfamily B, polypeptide 34 |
| Glyma.09G054600.1 | Ankyrin repeat family protein |
| Glyma.09G054700.1 | Ankyrin repeat family protein |
| Glyma.09G214100.1 | S-adenosyl-L-methionine-dependent methyltransferases superfamily protein |
| Glyma.09G277100.1 | Saposin B domain-containing protein |
| Glyma.09G280900.1 | Glutaredoxin-related |
| Glyma.10G029100.1 | 2-oxoglutarate (2OG) and Fe(II)-dependent oxygenase superfamily protein |
| Glyma.10G108200.1 | PIF1 helicase |
| Glyma.10G187200.1 | P-loop containing nucleoside triphosphate hydrolases superfamily protein |
| Glyma.10G250700.1 | Protein of unknown function (DUF3049) |
| Glyma.10G259900.1 | Thioredoxin domain-containing protein 9 homolog |
| Glyma.10G264100.1 | Heavy metal transport/detoxification superfamily protein |
| Glyma.10G267800.1 | MATE efflux family protein |
| Glyma.11G037100.1 | FAD/NAD(P)-binding oxidoreductase family protein |
| Glyma.11G044200.1 | Tubulin alpha-2 chain |
| Glyma.11G106600.1 | Actin depolymerizing factor 5 |
| Glyma.11G164700.1 | NAD(P)-binding Rossmann-fold superfamily protein |
| Glyma.11G249900.1 | Microtubule-associated protein 65-8 |
| Glyma.12G017100.1 | Polyketide cyclase/dehydrase and lipid transport superfamily protein |
| Glyma.12G156500.1 | Pentatricopeptide repeat (PPR) superfamily protein |
| Glyma.12G188200.1 | Histone deacetylase 8 |
| Glyma.12G191800.1 | Cellulose synthase-like B4 |
| Glyma.12G202400.1 | Protein phosphatase 2C family protein |
| Glyma.12G205900.1 | Tyrosine transaminase family protein |
| Glyma.13G077200.1 | Uncharacterized protein |
| Glyma.13G160100.1 | Raffinose synthase family protein |
| Glyma.13G174000.1 | Transcriptional factor B3 family protein / auxin-responsive factor AUX/IAA-related |
| Glyma.13G186600.1 | Zinc induced facilitator-like 1 |
| Glyma.13G217400.1 | Cytochrome P450, family 710, subfamily A, polypeptide 1 |
| Glyma.13G238600.1 | 3-ketoacyl-CoA synthase 10 |
| Glyma.13G257800.1 | Hydroxyproline-rich glycoprotein family protein |
| Glyma.13G327000.1 | Beta-galactosidase 8 |
| Glyma.14G056700.1 | Lateral organ boundaries (LOB) domain family protein |
| Glyma.14G058200.1 | Basic helix-loop-helix (bHLH) DNA-binding superfamily protein |
| Glyma.14G071600.1 | HXXXD-type acyl-transferase family protein |
| Glyma.14G080500.1 | Serine carboxypeptidase-like 40 |
| Glyma.14G087400.1 | NAD(P)-binding Rossmann-fold superfamily protein |
| Glyma.14G104400.1 | Peroxidase superfamily protein |
| Glyma.14G121700.1 | Myosin heavy chain-related protein |
| Glyma.14G127800.1 | Pleiotropic drug resistance 12 |
| Glyma.14G190900.1 | Phosphate transporter 4;3 |
| Glyma.14G194800.1 | PHD finger transcription factor, putative |
| Glyma.14G216200.1 | Walls Are Thin 1 |
| Glyma.14G224400.1 | Tetratricopeptide repeat (TPR)-like superfamily protein |
| Glyma.15G025400.1 | IQ-domain 8 |
| Glyma.15G078300.1 | NAC domain containing protein 73 |
| Glyma.15G209200.1 | Polygalacturonase inhibiting protein 1 |
| Glyma.15G214100.1 | Uncharacterized protein |
| Glyma.15G214200.1 | Uncharacterized protein |
| Glyma.15G219700.1 | Uncharacterized protein |
| Glyma.15G252200.1 | Glutathione S-transferase TAU 19 |
| Glyma.16G055400.1 | Uncharacterized protein |
| Glyma.16G056300.1 | Ferritin/ribonucleotide reductase-like family protein |
| Glyma.16G099300.1 | LMBR1-like membrane protein |
| Glyma.16G126300.1 | Regulatory particle non-ATPase 10 |
| Glyma.16G133200.1 | Basic helix-loop-helix (bHLH) DNA-binding superfamily protein |
| Glyma.16G195000.1 | Pentatricopeptide repeat (PPR) superfamily protein |
| Glyma.16G195700.1 | Tetratricopeptide repeat (TPR)-like superfamily protein |
| Glyma.17G033400.1 | High chlorophyll fluorescent 107 |
| Glyma.17G044500.1 | Plant invertase/pectin methylesterase inhibitor superfamily |
| Glyma.17G139700.1 | Bifunctional inhibitor/lipid-transfer protein/seed storage 2S albumin superfamily protein |
| Glyma.17G144300.1 | 2-oxoglutarate (2OG) and Fe(II)-dependent oxygenase superfamily protein |
| Glyma.17G150200.1 | Poly(U)-specific endoribonuclease |
| Glyma.17G162500.1 | Uncharacterized protein |
| Glyma.17G165600.1 | Zinc finger protein 7 |
| Glyma.17G172400.1 | Basic helix-loop-helix (bHLH) DNA-binding superfamily protein |
| Glyma.17G175400.1 | Calcium-binding EF-hand family protein |
| Glyma.17G177800.1 | Peroxidase superfamily protein |
| Glyma.17G178000.1 | Ribosomal protein L25/Gln-tRNA synthetase, anti-codon-binding domain |
| Glyma.17G179000.1 | Cleavage and polyadenylation specificity factor 160 |
| Glyma.17G194600.1 | Major facilitator superfamily protein |
| Glyma.18G003200.1 | Xyloglucan endotransglucosylase/hydrolase 16 |
| Glyma.18G007900.1 | Galactose oxidase/kelch repeat superfamily protein |
| Glyma.18G027500.1 | Protein of unknown function (DUF1313) |
| Glyma.18G073500.1 | Kinesin-like protein 1 |
| Glyma.18G103400.1 | Spermidine hydroxycinnamoyl transferase |
| Glyma.18G191900.1 | Copper transporter 1 |
| Glyma.18G195200.1 | Receptor like protein 13 |
| Glyma.18G243800.1 | Uncharacterized conserved protein ycf60 |
| Glyma.19G078900.1 | GTP binding Elongation factor Tu family protein |
| Glyma.20G013800.1 | Hydroxyproline-rich glycoprotein family protein |
| Glyma.20G152400.1 | Alpha-L-fucosidase 1 |
| Glyma.U029200.1 | Uncharacterized protein |
| Glyma.U030800.1 | GATA type zinc finger transcription factor family protein |
| Glyma.U031100.1 | Uncharacterized protein |
