# Supplementary material for: Transcriptomic evidence for the control of soybean root isoflavonoid content by regulation of overlapping phenylpropanoid pathways
Source: BMC Genomics. 2017 Jan 11;18:70. doi: 10.1186/s12864-016-3463-y (PMC5225596; doi:10.1186/s12864-016-3463-y)
Supplement: Additional file 7: — Table S3. List of genes downregulated in high (Conrad and AC Colombe) as compared with low (AC Glengarry and Pagoda) root isoflavonoid content cultivars. Differentially expressed genes (p < 0.05) in the four comparisons between high and low cultivars were analyzed for overlap (Fig. 2), generating a set of 104 candidates downregulated consistently in high root isoflavonoid cultivars. (DOCX 22 kb) [file 12864_2016_3463_MOESM7_ESM.docx]

**Table S3** List of genes downregulated in high (Conrad and AC Colombe) as compared with low (AC Glengarry and Pagoda) root isoflavonoid content cultivars. Differentially expressed genes (p<0.05) in the four comparisons between high and low cultivars were analyzed for overlap (Fig. 2), generating a set of 104 candidates downregulated consistently in high root isoflavonoid cultivars. These genes were annotated using the soybean database and have been compiled below:

| **Glyma identifier** | **Annotation (domain and motif description)** |
| --- | --- |
| Glyma.01G032400.1 | Disease resistance protein (TIR-NBS-LRR class), putative |
| Glyma.01G126600.1 | Disease resistance-responsive (dirigent-like protein) family protein |
| Glyma.02G028100.1 | Matrixin family protein |
| Glyma.02G064100.1 | Ribonuclease 1 |
| Glyma.02G064400.1 | Ribonuclease 1 |
| Glyma.02G157000.1 | Inorganic H pyrophosphatase family protein |
| Glyma.02G243400.1 | STELAR K+ outward rectifier |
| Glyma.03G037000.1 | LRR and NB-ARC domains-containing disease resistance protein |
| Glyma.03G044500.1 | Disease resistance-responsive (dirigent-like protein) family protein |
| Glyma.03G054800.1 | Receptor like protein 6 |
| Glyma.03G055400.1 | Pleckstrin homology (PH) domain superfamily protein |
| Glyma.03G058800.1 | Uncharacterized protein |
| Glyma.03G066800.1 | NAD(P)-linked oxidoreductase superfamily protein |
| Glyma.03G111400.1 | ARM repeat superfamily protein |
| Glyma.03G125500.1 | Serine carboxypeptidase-like 45 |
| Glyma.03G186300.1 | Phosphatidyl inositol monophosphate 5 kinase |
| Glyma.03G254300.1 | Chitinase A |
| Glyma.04G151500.1 | SNF7 family protein |
| Glyma.04G190400.1 | Leucine-rich repeat protein kinase family protein |
| Glyma.05G002500.1 | Glucuronokinase G |
| Glyma.05G057400.1 | Bi-functional inhibitor/lipid-transfer protein/seed storage 2S albumin superfamily protein |
| Glyma.05G069200.1 | Uncharacterized protein |
| Glyma.05G100100.1 | dsRNA-binding protein 2 |
| Glyma.05G100200.1 | Nucleotide-sugar transporter family protein |
| Glyma.05G125200.1 | FAD-binding Berberine family protein |
| Glyma.05G231800.1 | Aldehyde dehydrogenase 2C4 |
| Glyma.06G013600.1 | Phosphatidylinositol glycan, class X |
| Glyma.06G045600.1 | Ubiquitin system component Cue protein |
| Glyma.06G179200.1 | Raffinose synthase family protein |
| Glyma.06G250600.1 | Uncharacterized protein |
| Glyma.06G294100.1 | Ankyrin repeat family protein |
| Glyma.06G302700.1 | Peroxidase superfamily protein |
| Glyma.07G049400.1 | Pseudo-response regulator 5 |
| Glyma.07G078000.1 | NB-ARC domain-containing disease resistance protein |
| Glyma.07G147400.1 | Tetratricopeptide repeat (TPR)-like superfamily protein |
| Glyma.07G156900.1 | Chaperone DnaJ-domain superfamily protein |
| Glyma.07G157000.1 | Subtilase family protein |
| Glyma.07G162700.1 | Glycosyl hydrolase family protein |
| Glyma.07G186100.1 | D-aminoacid aminotransferase-like PLP-dependent enzymes superfamily protein |
| Glyma.08G062600.1 | Sulfite exporter TauE/SafE family protein |
| Glyma.08G066300.1 | F-box family protein with a domain of unknown function (DUF295) |
| Glyma.08G070000.1 | 2-oxoglutarate (2OG) and Fe(II)-dependent oxygenase superfamily protein |
| Glyma.08G111000.1 | Beta galactosidase 9 |
| Glyma.08G276200.1 | Uncharacterized protein |
| Glyma.09G049300.1 | Cytochrome P450, family 81, subfamily D, polypeptide 3 |
| Glyma.09G106100.1 | Uncharacterized protein |
| Glyma.09G238200.1 | NOD26-like intrinsic protein 4;2 |
| Glyma.09G271000.1 | Cytochrome P450, family 714, subfamily A, polypeptide 1 |
| Glyma.09G271900.1 | ZIP metal ion transporter family |
| Glyma.09G276900.1 | TATA binding protein 2 |
| Glyma.09G278700.1 | HEAT repeat ;WD domain, G-beta repeat protein protein |
| Glyma.09G279100.1 | Cytochrome P450, family 71, subfamily B, polypeptide 34 |
| Glyma.10G184900.1 | Ureidoglycolate amidohydrolase |
| Glyma.10G227700.1 | Chitinase A |
| Glyma.10G269200.1 | Uncharacterized protein |
| Glyma.10G270100.1 | Phosphatidic acid phosphatase 1 |
| Glyma.10G271400.1 | Protein kinase superfamily protein |
| Glyma.11G205400.1 | Cysteine-rich RLK (RECEPTOR-like protein kinase) 3 |
| Glyma.11G206400.1 | Cysteine-rich RLK (RECEPTOR-like protein kinase) 2 |
| Glyma.12G025600.1 | Serine carboxypeptidase-like 34 |
| Glyma.12G132400.1 | Disease resistance protein (TIR-NBS-LRR class), putative |
| Glyma.12G158300.1 | Small nuclear ribonucleoprotein family protein |
| Glyma.12G193600.1 | Galactosyltransferase family protein |
| Glyma.13G000200.1 | Methionine--tRNA ligase, putative / methionyl-tRNA synthetase, putative / MetRS, putative |
| Glyma.13G001500.1 | YELLOW STRIPE like 1 |
| Glyma.13G032900.1 | Protein kinase superfamily protein |
| Glyma.13G033400.1 | Protein kinase superfamily protein |
| Glyma.13G033900.1 | Suppressor of npr1-1 constitutive 4 |
| Glyma.13G072100.1 | Cytochrome P450 superfamily protein |
| Glyma.13G077700.1 | Wall-associated kinase 2 |
| Glyma.13G147400.1 | Uncharacterized protein |
| Glyma.13G167700.1 | Major facilitator superfamily protein |
| Glyma.13G167900.1 | Ribosome biogenesis regulatory protein (RRS1) family protein |
| Glyma.13G168700.1 | Formate dehydrogenase |
| Glyma.13G188700.1 | Uncharacterized protein |
| Glyma.13G272300.1 | Sodium/calcium exchanger family protein / calcium-binding EF hand family protein |
| Glyma.14G034100.1 | HXXXD-type acyl-transferase family protein |
| Glyma.14G058600.1 | 2-oxoglutarate (2OG) and Fe(II)-dependent oxygenase superfamily protein |
| Glyma.14G061400.1 | Nucleotide-diphospho-sugar transferases superfamily protein |
| Glyma.14G099200.1 | S-adenosyl-L-methionine-dependent methyltransferases superfamily protein |
| Glyma.14G116000.1 | Protein kinase superfamily protein |
| Glyma.14G205300.1 | NB-ARC domain-containing disease resistance protein |
| Glyma.15G031400.1 | Beta glucosidase 15 |
| Glyma.15G228500.1 | Uncharacterized protein |
| Glyma.15G229400.1 | Uncharacterized protein |
| Glyma.15G264300.1 | GDSL-motif lipase 5 |
| Glyma.16G002700.1 | Uncharacterized protein |
| Glyma.17G029200.1 | Protein phosphatase 2C family protein |
| Glyma.17G031800.1 | Acyl-activating enzyme 7 |
| Glyma.17G032000.1 | Secretion-associated RAS super family 2 |
| Glyma.17G134200.1 | Cytochrome P450, family 706, subfamily A, polypeptide 4 |
| Glyma.17G136400.1 | MATE efflux family protein |
| Glyma.17G171400.1 | Kinase interacting (KIP1-like) family protein |
| Glyma.17G171500.1 | Protein kinase superfamily protein |
| Glyma.17G173200.1 | Dihydroflavonol 4-reductase |
| Glyma.18G086200.1 | NB-ARC domain-containing disease resistance protein |
| Glyma.18G202800.1 | Acyl-CoA oxidase 4 |
| Glyma.18G239500.1 | Subtilisin-like serine endopeptidase family protein |
| Glyma.18G257800.1 | Leucine-rich repeat receptor-like protein kinase family protein |
| Glyma.19G029300.1 | UDP-Glycosyltransferase superfamily protein |
| Glyma.20G022300.1 | glycosyl hydrolase family 17 protein |
| Glyma.20G148900.1 | homocysteine S-methyltransferase 3 |
| Glyma.U000400.1 | APRATAXIN-like |
| Glyma.U000500.1 | APRATAXIN-like |
