# Supplementary material for: Transcriptomic evidence for the control of soybean root isoflavonoid content by regulation of overlapping phenylpropanoid pathways
Source: BMC Genomics. 2017 Jan 11;18:70. doi: 10.1186/s12864-016-3463-y (PMC5225596; doi:10.1186/s12864-016-3463-y)
Supplement: Additional file 8: — Table S4. List of genes upregulated in high (Conrad and AC Colombe) as compared with low (AC Glengarry and Pagoda) root isoflavonoid content cultivars. Highly differentially expressed genes (p < 0.001) in the four comparisons between high and low cultivars were analyzed for overlap (Fig. 2), generating a set of 29 candidates upregulated consistently in high root isoflavonoid cultivars. (DOCX 16 kb) [file 12864_2016_3463_MOESM8_ESM.docx]

**Table S4** List of genes upregulated in high (Conrad and AC Colombe) as compared with low (AC Glengarry and Pagoda) root isoflavonoid content cultivars. Highly differentially expressed genes (p<0.001) in the four comparisons between high and low cultivars were analyzed for overlap (Fig. 2), generating a set of 29 candidates upregulated consistently in high root isoflavonoid cultivars. These genes were annotated using the soybean database and have been compiled below:

| **Glyma identifier** | **Annotation (domain and motif description)** |
| --- | --- |
| Glyma.01G000800.1 | RNA recognition motif |
| Glyma.01G122300.1 | Glycine-rich protein |
| Glyma.03G065700.1 | GRAS family transcription factor |
| Glyma.03G067100.1 | Uncharacterized protein family (UPF0497) |
| Glyma.03G068900.1 | AtGCP3 interacting protein 1 |
| Glyma.03G070300.1 | Serine carboxypeptidase-like 19 |
| Glyma.04G110500.1 | Uncharacterized protein |
| Glyma.06G138700.1 | Ribosomal protein L6 family |
| Glyma.06G213600.1 | Histone H3 K4-specific methyltransferase SET7/9 family protein |
| Glyma.06G268600.1 | Disease resistance protein (TIR-NBS-LRR class), putative |
| Glyma.06G268700.1 | Disease resistance protein (TIR-NBS-LRR class), putative |
| Glyma.06G274200.1 | Ribosomal L5P family protein |
| Glyma.06G308400.1 | Alpha/beta-Hydrolases superfamily protein |
| Glyma.08G024800.1 | RING/U-box superfamily protein |
| Glyma.08G087100.1 | Thioredoxin O1 |
| Glyma.08G326700.1 | Plant transposase (Ptta/En/Spm family) |
| Glyma.09G054600.1 | Ankyrin repeat family protein |
| Glyma.10G029100.1 | 2-oxoglutarate (2OG) and Fe(II)-dependent oxygenase superfamily protein |
| Glyma.11G037100.1 | FAD/NAD(P)-binding oxidoreductase family protein |
| Glyma.12G188200.1 | Histone deacetylase 8 |
| Glyma.12G191800.1 | Cellulose synthase-like B4 |
| Glyma.14G056700.1 | Lateral organ boundaries (LOB) domain family protein |
| Glyma.15G214100.1 | Uncharacterized protein |
| Glyma.17G144300.1 | 2-oxoglutarate (2OG) and Fe(II)-dependent oxygenase superfamily protein |
| Glyma.17G165600.1 | Zinc finger protein 7 |
| Glyma.17G177800.1 | Peroxidase superfamily protein |
| Glyma.17G178000.1 | Ribosomal protein L25/Gln-tRNA synthetase, anti-codon-binding domain |
| Glyma.17G179000.1 | Cleavage and polyadenylation specificity factor 160 |
| Glyma.17G194600.1 | Major facilitator superfamily protein |
