# Supplementary material for: Transcriptomic evidence for the control of soybean root isoflavonoid content by regulation of overlapping phenylpropanoid pathways
Source: BMC Genomics. 2017 Jan 11;18:70. doi: 10.1186/s12864-016-3463-y (PMC5225596; doi:10.1186/s12864-016-3463-y)
Supplement: Additional file 9: — Table S5. List of genes downregulated in high (Conrad and AC Colombe) as compared with low (AC Glengarry and Pagoda) root isoflavonoid content cultivars. List of genes downregulated in high (Conrad and AC Colombe) as compared with low (AC Glengarry and Pagoda) root isoflavonoid content cultivars. Highly differentially expressed genes (p < 0.001) in the four comparisons between high and low cultivars were analyzed for overlap (Fig. 2), generating a set of 35 candidates downregulated consistently in high root isoflavonoid cultivars. These genes were annotated using the soybean database and have been compiled below. (DOCX 17 kb) [file 12864_2016_3463_MOESM9_ESM.docx]

**Table S5** List of genes downregulated in high (Conrad and AC Colombe) as compared with low (AC Glengarry and Pagoda) root isoflavonoid content cultivars. Highly differentially expressed genes (p<0.001) in the four comparisons between high and low cultivars were analyzed for overlap (Fig. 2), generating a set of 35 candidates downregulated consistently in high root isoflavonoid cultivars. These genes were annotated using the soybean database and have been compiled below:

| **Glyma identifier** | **Annotation (domain and motif description)** |
| --- | --- |
| Glyma.01G032400.1 | Disease resistance protein (TIR-NBS-LRR class), putative |
| Glyma.02G157000.1 | Inorganic H pyrophosphatase family protein |
| Glyma.03G037000.1 | LRR and NB-ARC domains-containing disease resistance protein |
| Glyma.03G055400.1 | Pleckstrin homology (PH) domain superfamily protein |
| Glyma.03G066800.1 | NAD(P)-linked oxidoreductase superfamily protein |
| Glyma.03G111400.1 | ARM repeat superfamily protein |
| Glyma.04G151500.1 | SNF7 family protein |
| Glyma.05G057400.1 | Bi-functional inhibitor/lipid-transfer protein/seed storage 2S albumin superfamily protein |
| Glyma.05G100100.1 | dsRNA-binding protein 2 |
| Glyma.05G100200.1 | Nucleotide-sugar transporter family protein |
| Glyma.06G179200.1 | Raffinose synthase family protein |
| Glyma.07G078000.1 | NB-ARC domain-containing disease resistance protein |
| Glyma.07G156900.1 | Chaperone DnaJ-domain superfamily protein |
| Glyma.07G157000.1 | Subtilase family protein |
| Glyma.07G162700.1 | Glycosyl hydrolase family protein |
| Glyma.08G070000.1 | 2-oxoglutarate (2OG) and Fe(II)-dependent oxygenase superfamily protein |
| Glyma.09G279100.1 | Cytochrome P450, family 71, subfamily B, polypeptide 34 |
| Glyma.10G184900.1 | Ureidoglycolate amidohydrolase |
| Glyma.10G269200.1 | Uncharacterized protein |
| Glyma.12G158300.1 | Small nuclear ribonucleoprotein family protein |
| Glyma.13G167700.1 | Major facilitator superfamily protein |
| Glyma.13G167900.1 | Ribosome biogenesis regulatory protein (RRS1) family protein |
| Glyma.13G272300.1 | Sodium/calcium exchanger family protein / calcium-binding EF hand family protein |
| Glyma.14G058600.1 | 2-oxoglutarate (2OG) and Fe(II)-dependent oxygenase superfamily protein |
| Glyma.14G061400.1 | Nucleotide-diphospho-sugar transferases superfamily protein |
| Glyma.14G205300.1 | NB-ARC domain-containing disease resistance protein |
| Glyma.15G031400.1 | Beta glucosidase 15 |
| Glyma.15G228500.1 | Uncharacterized protein |
| Glyma.17G031800.1 | Acyl-activating enzyme 7 |
| Glyma.17G171400.1 | Kinase interacting (KIP1-like) family protein |
| Glyma.17G171500.1 | Protein kinase superfamily protein |
| Glyma.17G173200.1 | Dihydroflavonol 4-reductase |
| Glyma.18G086200.1 | NB-ARC domain-containing disease resistance protein |
| Glyma.18G239500.1 | Subtilisin-like serine endopeptidase family protein |
| Glyma.U000400.1 | APRATAXIN-like |
