# Supplementary material for: Transcriptomic evidence for the control of soybean root isoflavonoid content by regulation of overlapping phenylpropanoid pathways
Source: BMC Genomics. 2017 Jan 11;18:70. doi: 10.1186/s12864-016-3463-y (PMC5225596; doi:10.1186/s12864-016-3463-y)
Supplement: Additional file 11: — Table S6. Genes upregulated in ‘high isoflavonoid’ cultivars (138 genes) were analyzed for overrepresentation of PANTHER GO-Slim classifications for: biological process, molecular function and cellular component. (DOCX 18 kb) [file 12864_2016_3463_MOESM11_ESM.docx]

**Table S6** Genes upregulated in ‘high isoflavonoid’ cultivars (138 genes) were analyzed for overrepresentation of PANTHER GO-Slim classifications for: biological process, molecular function and cellular component. The analysis was conducted with *Arabidopsis thaliana* homologs, against the reference database of that species, using the PANTHER Overrepresentation Test, (version 10.0; released 2015-05-15). The columns indicate the number of gene ids associated with a given classification: in the *Arabidopsis* database, the query list, and expected numbers based on the reference database. Using a binomial test of the query list against the expected values, fold enrichment, over- or under-representation (denoted by ‘+’ or ‘-’), and the p value are calculated. The table has been divided into the three classification systems, and sorted by order of ascending p value in each system.

|  | **Number of gene ids** | | |  |  |  |
| --- | --- | --- | --- | --- | --- | --- |
| [**PANTHER GO-Slim Biological Process**](http://pantherdb.org/tools/compareToRefList.jsp?sortOrder=1&sortList=categories&showAll=true) | ***Arabidopsis*** | [**Candidate List**](http://pantherdb.org/tools/compareToRefList.jsp?sortOrder=1&sortList=Client%20Text%20Box%20Input&sortField=pval&showAll=true) | **Expected** | **+/-** | **Fold Enrichment** | **P Value** |
| Cytokinesis (GO:0000910) | 66 | 2 | 0.3 | + | 6.68 | 3.66E-02 |
| Cellular component morphogenesis (GO:0032989) | 131 | 3 | 0.59 | + | 5.05 | 2.22E-02 |
| Anatomical structure morphogenesis (GO:0009653) | 131 | 3 | 0.59 | + | 5.05 | 2.22E-02 |
|  |  |  |  |  |  |  |
| **PANTHER GO-Slim Molecular Function** |  |  |  |  |  |  |
| Structural constituent of cytoskeleton (GO:0005200) | 393 | 6 | 1.78 | + | 3.37 | 9.33E-03 |
|  |  |  |  |  |  |  |
| **PANTHER GO-Slim Cellular Component** |  |  |  |  |  |  |
| Microtubule (GO:0005874) | 76 | 2 | 0.34 | + | 5.8 | 4.71E-02 |
| Cytoskeleton (GO:0005856) | 421 | 6 | 1.91 | + | 3.14 | 1.27E-02 |
