# Supplementary material for: Transcriptomic evidence for the control of soybean root isoflavonoid content by regulation of overlapping phenylpropanoid pathways
Source: BMC Genomics. 2017 Jan 11;18:70. doi: 10.1186/s12864-016-3463-y (PMC5225596; doi:10.1186/s12864-016-3463-y)
Supplement: Additional file 12: — Table S7. Genes downregulated in ‘high isoflavonoid’ cultivars (104 genes) were analyzed for overrepresentation of PANTHER GO-Slim classifications for: biological process, molecular function and cellular component. (DOCX 21 kb) [file 12864_2016_3463_MOESM12_ESM.docx]

**Table S7** Genes downregulated in ‘high isoflavonoid’ cultivars (104 genes) were analyzed for overrepresentation of PANTHER GO-Slim classifications for: biological process, molecular function and cellular component. The analysis was conducted with *Arabidopsis thaliana* homologs, against the reference database of that species, using the PANTHER Overrepresentation Test, (version 10.0; released 2015-05-15). The columns indicate the number of gene ids associated with a given classification: in the *Arabidopsis* database, the query list, and expected numbers based on the reference database. Using a binomial test of the query list against the expected values, fold enrichment, over- or under-representation (denoted by ‘+’ or ‘-’), and the p value are calculated. The table has been divided into the three classification systems, and sorted by order of ascending p value in each system.

|  | **Number of gene ids** | | |  |  |  |
| --- | --- | --- | --- | --- | --- | --- |
| [**PANTHER GO-Slim Biological Process**](http://pantherdb.org/tools/compareToRefList.jsp?sortOrder=1&sortList=categories&showAll=true) | ***Arabidopsis*** | [**Candidate List**](http://pantherdb.org/tools/compareToRefList.jsp?sortOrder=1&sortList=Client%20Text%20Box%20Input&sortField=pval&showAll=true) | **Expected** | **+/-** | **Fold Enrichment** | **P Value** |
| Unclassified (UNCLASSIFIED) | 16678 | 40 | 56.25 | - | 0.71 | 3.77E-04 |
| Metabolic process (GO:0008152) | 8659 | 43 | 29.21 | + | 1.47 | 1.77E-03 |
| Secondary metabolic process (GO:0019748) | 163 | 4 | 0.55 | + | 7.28 | 2.34E-03 |
| Biosynthetic process (GO:0009058) | 637 | 6 | 2.15 | + | 2.79 | 2.10E-02 |
| RNA localization (GO:0006403) | 68 | 2 | 0.23 | + | 8.72 | 2.24E-02 |
| Lipid metabolic process (GO:0006629) | 855 | 7 | 2.88 | + | 2.43 | 2.57E-02 |
| Nuclear transport (GO:0051169) | 88 | 2 | 0.3 | + | 6.74 | 3.60E-02 |
| Protein targeting (GO:0006605) | 100 | 2 | 0.34 | + | 5.93 | 4.53E-02 |
|  |  |  |  |  |  |  |
| **PANTHER GO-Slim Molecular Function** |  |  |  |  |  |  |
| Unclassified (UNCLASSIFIED) | 17570 | 43 | 59.26 | - | 0.73 | 3.21E-04 |
| Catalytic activity (GO:0003824) | 6641 | 37 | 22.4 | + | 1.65 | 5.29E-04 |
| Hydrolase activity, hydrolyzing O-glycosyl compounds (GO:0004553) | 65 | 3 | 0.22 | + | 13.68 | 1.45E-03 |
| Oxidoreductase activity (GO:0016491) | 1224 | 11 | 4.13 | + | 2.66 | 2.77E-03 |
| Hydrolase activity (GO:0016787) | 2046 | 13 | 6.9 | + | 1.88 | 1.97E-02 |
| Serine-type peptidase activity (GO:0008236) | 367 | 4 | 1.24 | + | 3.23 | 3.60E-02 |
| Phosphoric diester hydrolase activity (GO:0008081) | 14 | 1 | 0.05 | + | 21.18 | 4.61E-02 |
|  |  |  |  |  |  |  |
| **PANTHER GO-Slim Cellular Component** |  |  |  |  |  |  |
| External encapsulating structure (GO:0030312) | 68 | 3 | 0.23 | + | 13.08 | 1.65E-03 |
| Plasma membrane (GO:0005886) | 148 | 3 | 0.5 | + | 6.01 | 1.40E-02 |
| Integral to membrane (GO:0016021) | 87 | 2 | 0.29 | + | 6.82 | 3.52E-02 |
