# Supplementary material for: Transcriptomic evidence for the control of soybean root isoflavonoid content by regulation of overlapping phenylpropanoid pathways
Source: BMC Genomics. 2017 Jan 11;18:70. doi: 10.1186/s12864-016-3463-y (PMC5225596; doi:10.1186/s12864-016-3463-y)
Supplement: Additional file 13: — Table S8. Genes upregulated in ‘high isoflavonoid’ cultivars (138 genes) were analyzed for pathway enrichment. (DOCX 19 kb) [file 12864_2016_3463_MOESM13_ESM.docx]

**Table S8** Genes upregulated in ‘high isoflavonoid’ cultivars (138 genes) were analyzed for pathway enrichment. This was performed using the PhytoMine tool, ‘Pathway Enrichment’ (accessed 2016-05-01; Phytozome version 11). The *Glycine max* database was selected as reference. Benjamini-Hochberg statistical analysis was used to generate p values for the pathway categories. The columns indicate: pathway name, number of genes in the candidate list matching the pathway, the p-value (determined by a binomial test of the number of matches against the expected values), and the Glyma identifiers corresponding to the matches. The table has been sorted by order of ascending p value.

| **Pathway** | **Matches** | **p-Value** | **Glyma identifiers** |
| --- | --- | --- | --- |
| Phenylalanine metabolism | 4 | 0.048 | Glyma.06G178400, Glyma.12G205900,  Glyma.14G104400, Glyma.17G177800 |
| Isoquinoline alkaloid biosynthesis | 2 | 0.08 | Glyma.06G178400, Glyma.12G205900 |
| Tropane, piperidine and pyridine alkaloid biosynthesis | 2 | 0.08 | Glyma.06G178400, Glyma.12G205900 |
| Tyrosine metabolism | 2 | 0.18 | Glyma.06G178400, Glyma.12G205900 |
| Phenylpropanoid biosynthesis | 3 | 0.44 | Glyma.03G070300, Glyma.14G104400,  Glyma.17G177800 |
| mRNA surveillance pathway | 2 | 0.97 | Glyma.01G000800, Glyma.17G179000 |
| Pentose and glucuronate interconversions | 1 | 1 | Glyma.17G044500 |
| Steroid biosynthesis | 1 | 1 | Glyma.13G217400 |
| Ubiquinone and other terpenoid-quinone biosynthesis | 1 | 1 | Glyma.12G205900 |
| Purine metabolism | 1 | 1 | Glyma.16G056300 |
| Pyrimidine metabolism | 1 | 1 | Glyma.16G056300 |
| Glycine, serine and threonine metabolism | 1 | 1 | Glyma.06G178400 |
| Cysteine and methionine metabolism | 1 | 1 | Glyma.12G205900 |
| Phenylalanine, tyrosine and tryptophan biosynthesis | 1 | 1 | Glyma.12G205900 |
| beta-Alanine metabolism | 1 | 1 | Glyma.06G178400 |
| Glutathione metabolism | 1 | 1 | Glyma.16G056300 |
| Starch and sucrose metabolism | 1 | 1 | Glyma.17G044500 |
| Limonene and pinene degradation | 1 | 1 | Glyma.08G326900 |
| Stilbenoid, diarylheptanoid and gingerol biosynthesis | 1 | 1 | Glyma.08G326900 |
| Ribosome | 1 | 1 | Glyma.06G138700 |
| RNA transport | 2 | 1 | Glyma.01G000800, Glyma.19G078900 |
| RNA degradation | 1 | 1 | Glyma.01G000800 |
| Proteasome | 1 | 1 | Glyma.16G126300 |
| Plant hormone signal transduction | 1 | 1 | Glyma.13G174000 |
