# Supplementary material for: Transcriptomic evidence for the control of soybean root isoflavonoid content by regulation of overlapping phenylpropanoid pathways
Source: BMC Genomics. 2017 Jan 11;18:70. doi: 10.1186/s12864-016-3463-y (PMC5225596; doi:10.1186/s12864-016-3463-y)
Supplement: Additional file 14: — Table S9. Genes downregulated in ‘high isoflavonoid’ cultivars (104 genes) were analyzed for pathway enrichment. (DOCX 20 kb) [file 12864_2016_3463_MOESM14_ESM.docx]

**Table S9** Genes downregulated in ‘high isoflavonoid’ cultivars (104 genes) were analyzed for pathway enrichment. This was performed using the PhytoMine tool, ‘Pathway Enrichment’ (accessed 2016-05-01; Phytozome version 11). The *Glycine max* database was selected as reference. Benjamini-Hochberg statistical analysis was used to generate p values for the pathway categories. The columns indicate: pathway name, number of genes in the candidate list matching the pathway, the p-value (determined by a binomial test of the number of matches against the expected values), and the Glyma identifiers corresponding to the matches. The table has been sorted by order of ascending p value.

| **Pathway** | **Matches** | **p-Value** | **Glyma identifiers** |
| --- | --- | --- | --- |
| Fatty acid degradation | 1 | 1 | Glyma.18G202800 |
| Oxidative phosphorylation | 1 | 1 | Glyma.02G157000 |
| Cysteine and methionine metabolism | 2 | 1 | Glyma.14G099200, Glyma.20G148900 |
| Valine, leucine and isoleucine degradation | 1 | 1 | Glyma.07G186100 |
| Valine, leucine and isoleucine biosynthesis | 1 | 1 | Glyma.07G186100 |
| Arginine and proline metabolism | 1 | 1 | Glyma.14G099200 |
| Phenylalanine metabolism | 1 | 1 | Glyma.06G302700 |
| beta-Alanine metabolism | 1 | 1 | Glyma.14G099200 |
| Selenocompound metabolism | 1 | 1 | Glyma.13G000200 |
| Cyanoamino acid metabolism | 1 | 1 | Glyma.15G031400 |
| Glutathione metabolism | 1 | 1 | Glyma.14G099200 |
| Starch and sucrose metabolism | 1 | 1 | Glyma.15G031400 |
| Amino sugar and nucleotide sugar metabolism | 2 | 1 | Glyma.03G254300, Glyma.10G227700 |
| Inositol phosphate metabolism | 1 | 1 | Glyma.03G186300 |
| Glycosylphosphatidylinositol(GPI)-anchor biosynthesis | 1 | 1 | Glyma.06G013600 |
| alpha-Linolenic acid metabolism | 1 | 1 | Glyma.18G202800 |
| Glyoxylate and dicarboxylate metabolism | 1 | 1 | Glyma.13G168700 |
| Pantothenate and CoA biosynthesis | 1 | 1 | Glyma.07G186100 |
| Limonene and pinene degradation | 2 | 1 | Glyma.09G049300, Glyma.09G279100 |
| Phenylpropanoid biosynthesis | 3 | 1 | Glyma.05G231800, Glyma.06G302700,  Glyma.15G031400 |
| Flavonoid biosynthesis | 2 | 1 | Glyma.13G072100, Glyma.17G173200 |
| Flavone and flavonol biosynthesis | 1 | 1 | Glyma.13G072100 |
| Stilbenoid, diarylheptanoid and gingerol biosynthesis | 2 | 1 | Glyma.09G049300, Glyma.09G279100 |
| Aminoacyl-tRNA biosynthesis | 1 | 1 | Glyma.13G000200 |
| Biosynthesis of unsaturated fatty acids | 1 | 1 | Glyma.18G202800 |
| Basal transcription factors | 1 | 1 | Glyma.09G276900 |
| Spliceosome | 1 | 1 | Glyma.12G158300 |
| Phosphatidylinositol signaling system | 1 | 1 | Glyma.03G186300 |
| Endocytosis | 2 | 1 | Glyma.03G186300, Glyma.04G151500 |
| Peroxisome | 1 | 1 | Glyma.18G202800 |
| Plant-pathogen interaction | 2 | 1 | Glyma.07G078000, Glyma.18G086200 |
| Circadian rhythm - plant | 1 | 1 | Glyma.07G049400 |
