# Supplementary material for: Transcriptomic evidence for the control of soybean root isoflavonoid content by regulation of overlapping phenylpropanoid pathways
Source: BMC Genomics. 2017 Jan 11;18:70. doi: 10.1186/s12864-016-3463-y (PMC5225596; doi:10.1186/s12864-016-3463-y)
Supplement: Additional file 15: — Table S10. Genes annotated with disease or stress response and upregulated in ‘high isoflavonoid’ cultivars. (DOCX 18 kb) [file 12864_2016_3463_MOESM15_ESM.docx]

**Table S10** Genes annotated for disease or stress response and upregulated in ‘high isoflavonoid’ cultivars. This was performed using the TAIR (The Arabidopsis Information Resource, http://arabidopsis.org; accessed 2016-10-01). The columns indicate: Glyma identifiers and the corresponding transcript description.

| **Glyma identifier** | **Transcript description** |
| --- | --- |
| Glyma.14G127800 | ABC Transporter; ABA transport and lead resistance |
| Glyma.10G264100 | Heavy metal transport/detoxification |
| Glyma.17G177800 | Peroxidase superfamily protein |
| Glyma.11G044200 | Alpha-2,4 Tubulin |
| Glyma.01G127400 | Disease resistance-responsive (dirigent-like protein) |
| Glyma.14G216200 | Plant drug/metabolite exporter |
| Glyma.15G252200 | Glutathione transferase |
| Glyma.14G104400 | Peroxidase superfamily protein |
| Glyma.13G238600 | 3-Ketoacyl-COA synthase 10 |
| Glyma.08G087100 | Thioredoxin |
| Glyma.03G069100 | Cupin superfamily; resistance to Ralstonia solanaceae |
| Glyma.03G047000 | LRR and NB-ARC domains-containing disease resistance protein |
| Glyma.01G118900 | LRR-RLK protein disease resistance protein |
| Glyma.06G268700 | Disease resistance protein (TIR-NBS-LRR class) |
| Glyma.16G056300 | Ribonucleotide reductase (RNR) small subunit; DNA damage repair |
| Glyma.10G108200 | DNA helicase homolog PIF1 |
| Glyma.17G139700 | AZI family of lipid transfer proteins |
| Glyma.14G087400 | Progesterone-5beta-reductase-like protein |
| Glyma.06G134900 | Hsp21; chloroplast located small heat shock protein |
| Glyma.17G172400 | bHLH transcription factor; seed development and response |
| Glyma.16G126300 | Regulatory particle non-ATPase subunit of 26S proteasome |
| Glyma.15G209200 | Polygalacturonase inhibiting protein; defense response |
| Glyma.06G313600 | Zinc induced facilitator-like 1; tetracycline:hydrogen antiporter activity |
| Glyma.06G268600 | Disease resistance protein (TIR-NBS-LRR class) |
| Glyma.13G160100 | Glycosyl hydrolase family 36; light stress |
| Glyma.12G017100 | Polyketide cyclase/dehydrase and lipid transport |
| Glyma.08G062800 | BAK1-interacting receptor-like kinase; disease resistance signalling |
