# Supplementary material for: Transcriptomic evidence for the control of soybean root isoflavonoid content by regulation of overlapping phenylpropanoid pathways
Source: BMC Genomics. 2017 Jan 11;18:70. doi: 10.1186/s12864-016-3463-y (PMC5225596; doi:10.1186/s12864-016-3463-y)
Supplement: Additional file 16: — Table S11. Genes annotated for transcriptional regulation and upregulated in ‘high isoflavonoid’ cultivars. (DOCX 16 kb) [file 12864_2016_3463_MOESM16_ESM.docx]

**Table S11** Genes annotated for transcriptional regulation and upregulated in ‘high isoflavonoid’ cultivars. This was performed using the TAIR (The Arabidopsis Information Resource, http://arabidopsis.org; accessed 2016-10-01). The columns indicate: Glyma identifiers and the corresponding transcript description.

| **Glyma identifier** | **Transcript description** |
| --- | --- |
| Glyma.12G188200 | histone deacetylase 8 |
| Glyma.05G100900 | zinc finger protein |
| Glyma.02G008200 | RAD-like 6 (RL6) |
| Glyma.06G027000 | B-box zinc finger transcription factor BBX21 |
| Glyma.04G151000 | MYB family of transcriptional regulators |
| Glyma.07G181600 | Agamous-like 22, Flowering Arabidopsis QTL1 |
| Glyma.13G174000 | Auxin response transcription factor, ETT |
| Glyma.03G065700 | GRAS family transcription factor |
| Glyma.14G058200 | basic helix-loop-helix (bHLH) DNA-binding superfamily protein |
| Glyma.15G078300 | NAC domain containing protein 73 |
| Glyma.17G172400 | bHLH protein family |
| Glyma.14G194800 | PHD finger transcription factor |
